# Concordance of positive, negative and disorganised psychotic syndromes in five twin samples

**DOI:** 10.1192/bjo.2026.11017

**Published:** 2026-04-01

**Authors:** Alastair G. Cardno, E. Jane Marshall, Timothea Toulopoulou, Eugenia Kravariti, Edward J. Pepper, Frühling V. Rijsdijk, Judith Allardyce, Robin M. Murray, Evangelos Vassos

**Affiliations:** Division of Psychological and Social Medicine, University of Leeds, Leeds, UK; Addictions Clinical Academic Group, South London and Maudsley NHS Foundation Trust, London, UK; Department of Psychology, National Magnetic Resonance Research Center (UMRAM) & Aysel Sabuncu Brain Research Center (ASBAM), Bilkent University, Ankara, Turkey; Department of Psychiatry, School of Medicine, National and Kapodistrian University of Athens, Athens, Greece; Department of Psychiatry, Icahn School of Medicine at Mount Sinai, New York, USA; Department of Psychosis Studies, Institute of Psychiatry, Psychology and Neuroscience, King’s College London, London, UK; Child and Adolescent Mental Health Service (CAMHS), Bradford District Care NHS Foundation Trust, Bradford, UK; Psychology Department, Anton de Kom University of Suriname, Paramaribo, Suriname; Social Genetic and Developmental Psychiatry (SGDP) Centre, Institute of Psychiatry, Psychology and Neuroscience, King’s College London, London, UK; Centre for Clinical Brain Sciences, University of Edinburgh, Edinburgh, UK; Centre for Neuropsychiatric Genetics and Genomics, Cardiff University, Cardiff, UK; National Institute for Health and Care Research (NIHR) Maudsley Biomedical Research Centre (BRC), South London and Maudsley NHS Foundation Trust and King’s College London, London, UK

**Keywords:** Schizophrenia, psychosis, twin, concordance, heritability

## Abstract

**Background:**

Schizophrenia and psychosis have high twin heritability (approximately 80%), but these general estimates may hide aetiological variation. This can be investigated by using syndromes based on key psychotic symptom combinations.

**Aims:**

To investigate concordance, and heritability where calculable, of psychotic syndromes in multiple schizophrenia and psychosis twin samples.

**Method:**

We investigated concordance for positive, negative and disorganised psychotic syndromes, based on lifetime symptom ratings, in three classical schizophrenia twin samples (Fischer, Kringlen and Slater) and two psychosis samples (Maudsley register and non-register), the first four being systematically ascertained (total 317 monozygotic and 145 dizygotic probandwise pairs). We assessed concordance differences with logistic regression in generalised linear mixed models, and heritability from twin-modelling in the Maudsley register sample.

**Results:**

The positive syndrome, comprising delusions plus hallucinations, had 37.7–41.4% monozygotic and 6.0–6.3% dizygotic concordance, and heritability of 0.81 or 81% (95% CI 0.58–0.88), with similar results for negative and disorganised syndromes. In the systematically ascertained samples, delusions and hallucinations occurring without disorganised symptoms had nominally lower monozygotic twin concordance than when disorganised symptoms were also present (in the three schizophrenia samples: 89 pairs, odds ratio 3.47 (95% CI 1.04–1.54), *p* = 0.043; and the Maudsley register psychosis sample: 70 pairs, odds ratio 7.68 (95% CI 1.49–39.70), *p* = 0.016).

**Conclusions:**

In schizophrenia and psychosis, the syndrome of delusions plus hallucinations has high twin heritability overall. Positive symptoms without disorganised symptoms may indicate relatively high environmental influences, and positive symptoms with disorganised symptoms, relatively high familial and probably genetic influences, but further confirmation is needed.

From a top-down perspective, schizophrenia and psychosis (any psychotic disorder) have high twin heritability (approximately 80%),^1–8^ but within these broad clinical categories there are considerable differences in symptoms between individuals.^9^ And from a bottom-up perspective, psychotic symptoms are not randomly distributed, as certain symptoms tend to cluster together.^10,11^ This has led to the complementary middle perspective of psychotic symptom dimensions, where the pattern of co-occurrence of broadly defined psychotic symptoms can be summarised by factor analysis into three main dimensions: positive (delusions and hallucinations), negative (restricted speech and affect, reduced motivation and socialisation) and disorganised (formal thought disorder, incongruous/inappropriate affect, and bizarre behaviour).^12^ Subdivisions or merging of dimensions can also occur, depending on which symptoms are included.^13,14^ Affective symptoms^15^ and cognitive abilities^16^ are also sometimes included in analyses, and load on their own additional dimensions.

Among individuals with psychotic disorders, there are some associations with developmental and clinical factors that the three psychotic symptom dimensions share in common, e.g. higher levels of symptoms on all three dimensions (i.e. where the core symptoms co-occur) are associated with lower premorbid sociability and never marrying or cohabiting.^17^ There are also relatively distinct associations that may reflect some aetiological differences, e.g. positive symptoms are most associated with childhood adversity,^18,19^ ethnic minority status,^15,17^ cannabis use^20,21^ and better response to antipsychotic medication;^9^ negative symptoms are most associated with male gender and chronic illness course;^17^ and disorganised symptoms are most associated with substantive heritability.^4^ Negative and disorganised symptoms are also more associated with early age at onset^17^ and cognitive impairment^22^ than positive symptoms.

To date, investigations of psychotic symptom dimensions have focused on factors influencing variation among affected individuals or pairs of affected relatives (case-only analyses). In the current study, we extended this by defining psychotic symptom dimensions as syndromes in their own right, which allowed the absence or presence of the syndromes to be assessed in relatives, and hence investigation of factors influencing risk or liability to the syndromes in the population.

These psychotic syndromes differ from traditional schizophrenia subtypes because they are non-exclusive: it is possible to investigate general syndromes, such as a positive syndrome comprising delusions and hallucinations, and also subdivisions based on co-occurrence of syndromes, such as a positive syndrome co-occurring with negative symptoms. This gives flexibility to assess similarities and differences in risk factors or treatment effects across a range of syndrome configurations, as samples sizes allow, and to contribute to refining clinical psychosis phenotypes for research and clinical practice.

A key aspect of understanding phenotypes is to investigate the extent of familial influences (comprising genetic, or heritability (*h* ^2^), and common/shared-environmental (*c* ^2^) components) and individual-specific/non-shared environmental influences (*e* ^2^).^23^ This can be done in twin studies, subsequently linking with other types of research including investigations of particular genetic and environmental risk factors contributing to the *h* ^2^, *c* ^2^ and *e* ^2^ effects identified by twin studies.

## Study aims

We aimed to investigate familial/genetic and environmental influences on positive, negative and disorganised psychotic syndromes and subsyndromes in multiple schizophrenia and psychosis twin samples.

## Study hypotheses

First, as the positive, negative and disorganised syndromes comprised most of the core symptoms whose co-occurrence underpins commonly used diagnostic criteria for schizophrenia, and there is strong evidence for substantial familial and genetic influences on operationally defined schizophrenia, along with some individual-specific environmental influences, and little or no shared environmental influences,^1,2,6^ we hypothesised that there would be a similar pattern of influences on the general positive, negative and disorganised syndromes.

Second, as the relatively distinct influences on positive psychotic symptoms tend to be predominantly environmental (e.g. childhood adversity,^18,19^ ethnic minority status^15,17^ and cannabis use^20,21^), whereas higher levels of disorganised and/or negative symptoms are associated with higher heritability and genetic loading for schizophrenia, including polygenic risk scores,^14,16,24,25^ we hypothesised that delusions and hallucinations without negative or disorganised symptoms would have higher levels of environmental influences and lower levels of genetic influences, compared with the positive syndrome co-occurring with negative or disorganised symptoms.

## Method

### Samples

The study was based on three classical schizophrenia twin samples (investigated by Fischer,^26^ Kringlen^27,28^ and Slater^29^) and two psychosis samples^17^ (Maudsley register^1^ and Maudsley non-register^30,31^).

The schizophrenia samples were systematically ascertained from Scandinavian national twin registers linked with clinical registers (Fischer and Kringlen) or from mental hospitals in and around London, UK (Slater), and assessed by personal interviews and clinical case-record reviews, which were compiled into published case histories for the monozygotic twins.

The Maudsley register psychosis sample was systematically ascertained from the Maudsley and Bethlam Royal Hospitals’ clinical twin register in London, and assessed by research interviews^32,33^ and case-record reviews.

The Maudsley non-register psychosis sample was based on the combined Maudsley schizophrenia and bipolar twin study samples, ascertained non-systematically from clinicians and support groups in the UK, and assessed by research interviews^33–35^ and case-record reviews.

There was no overlap of twins in the final samples analysed in the current study.

Further information is given in Table 1, Supplementary Table 1 and Supplementary Method available at https://doi.org/10.1192/bjo.2026.11017.

**Table 1:** Characteristics of the five twin samples

Characteristic	Schizophrenia samples			Psychosis samples	
	Fischer schizophrenia sample	Kringlen schizophrenia sample	Slater schizophrenia sample	Maudsley register psychosis sample	Maudsley non-register psychosis sample
Setting	Denmark	Norway	London, UK	London, UK	UK
Ascertainment	Systematic – from national twin register linked with central psychiatric register	Systematic – from national twin register linked with central register of psychosis	Systematic – from standing population of mental hospitals, and in-patient and out-patient admissions	Systematic – from clinical twin register based on local in-patient, day patient and out-patient admissions	Non-systematic – from clinicians and support groups nationally
Number of probandwise pairs with sufficient information for analysis and proband had a psychotic disorder^a^	Monozygotic = 25	Monozygotic = 66	Monozygotic = 35	Monozygotic = 106 Same sex dizygotic = 118	Monozygotic = 85 Same sex dizygotic = 25 Opposite sex dizygotic = 2
% male gender	44.0	54.5	31.4	53.6	49.1
% White ethnicity	100	100	100	87.9	97.2 (6 missing)
% probands with DSM-III-R/IV main lifetime diagnosis of schizophrenia^b^	96.0	60.6	65.7	43.3	60.7
% with chronic illness course (from OPCRIT item 90 score 4 or 5)	80.0	41.3 (3 missing)	74.3	30.4 (5 missing)	31.3 (24 missing)
Year of birth	1878–1916	1901–1930	1861–1916	1887–1986	1940–1984
Age at onset of proband, mean (s.d.)^c^	32.7 (8.9) years	27.8 (8.2) years	29.9 (10.0) years	26.2 (11.1) years	21.0 (6.1) years
	range 22–54 years	range 17–47 years (2 with no specified age at onset)	range 9–51 years	range 7–67 years	range 5–50 years (1 missing)
Age of co-twin at last information, mean (s.d.)	59.0 (16.5) years	45.8 (10.0) years	42.6 (11.8) years	46.5 (15.4) years	37.0 (11.5) years
	range 18–87 years	range 22–64 years	range 23–71 years	range 15–88 years	range 20–61 years
Monozygotic probands:					
Positive symptom dimension scores, score: *n* (%)	0: 0 (0.0)	0: 7 (10.6)	0: 2 (5.7)	0: 5 (4.7)	0: 12 (14.1)
	1: 4 (16.0)	1: 16 (24.2)	1: 8 (22.9)	1: 31 (29.2)	1: 12 (14.1)
	2: 21 (84.0)	2: 43 (65.2)	2: 25 (71.4)	2: 70 (66.0)	2: 61 (71.8)
Negative symptom dimension scores, score: *n* (%)	0: 7 (28.0)	0: 26 (39.4)	0: 12 (34.3)	0: 54 (50.9)	0: 44 (51.8)
	1: 10 (40.0)	1: 13 (19.7)	1: 7 (20.0)	1: 40 (37.7)	1: 20 (23.5)
	2: 8 (32.0)	2: 27 (40.9)	2: 16 (45.7)	2: 12 (11.3)	2: 21 (24.7)
Disorganised symptom dimension scores, score: *n* (%)	0: 10 (40.0)	0: 33 (50.0)	0: 11 (31.4)	0: 49 (46.2)	0: 61 (71.8)
	1: 12 (48.0)	1: 24 (36.4)	1: 5 (14.3)	1: 33 (31.1)	1: 19 (22.4)
	2: 3 (12.0)	2: 9 (13.6)	2: 19 (54.3)	2: 24 (22.6)	2: 5 (5.9)
Dizygotic probands:					
Positive symptom dimension scores, score: *n* (%)				0: 4 (3.4)	0: 3 (11.1)
				1: 31 (26.3)	1: 8 (29.6)
				2: 83 (70.3)	2: 16 (59.3)
Negative symptom dimension scores, score: *n* (%)				0: 66 (55.9)	0: 17 (63.0)
				1: 29 (24.6)	1: 8 (29.6)
				2: 23 (19.5)	2: 2 (7.4)
Disorganised symptom dimension scores, score: *n* (%)				0: 53 (44.9)	0: 20 (74.1)
				1: 38 (32.2)	1: 6 (22.2)
				2: 27 (22.9)	2: 1 (3.7)

OPCRIT, Operational Criteria Checklist.

a. In schizophrenia samples, psychotic disorder defined as presence of at least one positive, negative or disorganised symptom. In psychosis samples, presence of manic or hypomanic episode also included.

b. Operational schizophrenia diagnoses – DSM-III-R in Maudsley register sample, DSM-IV in the other samples.

c. Age at onset as assessed by original author in schizophrenia samples, and age at first contact with mental health services in psychosis samples.

The schizophrenia and Maudsley register studies were established before the Declaration of Helsinki and research ethics committees, but were based on consistent principles. The Maudsley non-register study had approval from the UK Multicentre National Health Service (NHS) Research Ethics Committee and the Ethics Committee of the Institute of Psychiatry, Psychology and Neuroscience, King’s College London; all participants gave written informed consent.

### Defining psychotic syndromes

Psychotic syndromes were derived from psychotic symptom dimensions,^10–12^ based on lifetime symptom ratings obtained using the Operational Criteria Checklist (OPCRIT).^36^ They were defined as psychotic disorders with the following symptom combinations:

- narrow positive syndrome comprising any delusion/thought interference plus any hallucination (abbreviated as Pos = 2);
- broad negative syndrome comprising restricted affect and/or poverty of speech (Neg = 1 or 2), and narrow negative syndrome comprising both of these symptoms (Neg = 2);
- broad disorganised syndrome comprising positive formal thought disorder and/or inappropriate affect (Dis = 1 or 2), and narrow disorganised syndrome comprising both of these symptoms (Dis = 2).

In summary, the positive psychotic syndrome was composed of delusions and hallucinations, the negative syndromes of restricted affect and poverty of speech, and the disorganised syndromes of positive formal thought disorder and inappropriate affect. The narrow definitions of syndromes were rated as present if both core symptoms were present; and broad definitions were rated as present if either or both core symptoms were present; hence, the narrow syndromes were nested within the broad syndromes.

There were insufficient twin probands with less than two of the positive symptoms (Table 1) to include a broad positive syndrome. Additionally, it is similar to the phenotype of any psychotic disorder, which we have previously analysed in the Maudsley register sample.^4^

See also Supplementary Table 2 and Supplementary Method for further information.

### Analysis

#### Characteristics of the samples

We tabulated general characteristics of the samples and associations of syndromes with clinical and developmental variables in probands, to give context to the samples and phenotypes, and as a check for inconsistencies that might indicate errors in the data.

#### Concordance for psychotic syndromes

An overview of the twin concordance analysis plan is given in Supplementary Table 3, with further details in Supplementary Method.

We tabulated the probandwise concordance for each psychotic syndrome in monozygotic pairs and also dizygotic pairs, where applicable. We investigated differences between monozygotic and dizygotic concordances, including using zygosity as a predictor of concordance in logistic regression analysis.

#### Heritability of psychotic syndromes

Only the Maudsley register sample was suitable for twin modelling because it was a systematically-ascertained incident sample including both monozygotic and dizygotic pairs, and the lifetime morbid risk of syndromes could be estimated, in this case by extrapolation from local clinical case register and census data.^1^ In this sample, we calculated monozygotic and dizygotic tetrachoric correlations for each psychotic syndrome, which are based on the probandwise concordance and lifetime morbid risk of the phenotype within a liability-threshold model. We then estimated heritability within an ACE model, comprising additive genetic (*a* ^2^ or *h* ^2^), common/shared environmental (*c* ^2^) and individual-specific/non-shared (*e* ^2^) environmental influences.^23^

#### Monozygotic concordance for narrow positive syndrome in absence or presence of other syndromes

We investigated whether monozygotic concordance for the narrow positive syndrome was predicted by the absence versus presence of negative or disorganised syndromes also occurring in probands, using logistic regression analysis.

#### Concordance and heritability of narrow positive psychotic subsyndromes

We calculated monozygotic and dizygotic concordances, tetrachoric correlations and estimated heritability for narrow positive psychotic subsyndromes (e.g. among pairs where probands had the narrow positive syndrome without negative symptoms (Pos = 2 and Neg = 0), we investigated concordance for the same subsyndrome in co-twins), using the same methods as for the general psychotic syndromes.

#### Sensitivity analysis in twins with DSM schizophrenia, including combined samples analysis

Finally, we tabulated monozygotic concordance for the main psychotic syndromes and narrow positive subsyndromes, restricted to DSM schizophrenia (DSM-III-R^37^ in the Maudsley register sample and DSM-IV^38^ in the other samples) rather than psychotic disorder, to optimise homogeneity of diagnostic context across samples (there were no dizygotic pairs concordant for DSM schizophrenia to include in analysis). This included analysis of the four systematically ascertained samples combined (the three schizophrenia samples and the Maudsley register psychosis sample), and of all five samples combined.

We used SPSS version 29 for macOS (IBM, Armonk, New York, USA; https://www.ibm.com/spss) for tabulating concordances and logistic regression analysis, OpenEpi version 3.01 for macOS (https://openepi.com) to calculate Wald (normal approximation) based 95% confidence intervals around concordances, and Open Mx in R version 4.0.2 for macOS (https://openmx.ssri.psu.edu) for calculating tetrachoric correlations and twin modelling to estimate heritability.

We conducted logistic regression analyses of twin concordance within generalised linear mixed models, adjusted for gender and age of co-twin at last information included as potential confounders, with sample and twin pair modelled as random effects, where applicable.

We defined statistically significant results as those with 95% confidence intervals not overlapping the relevant null value. To evaluate the robustness of results, we focused on effect sizes, width of 95% confidence intervals, consistency of independent results between samples and results of combined samples analysis. If there were discrepancies between samples, the results for systematically ascertained samples took precedence.

In the logistic regression analysis predicting concordance for the narrow positive psychotic syndrome in monozygotic twin pairs according to the absence/presence of negative or disorganised syndromes also occurring in probands, we made an additional adjustment for multiple statistical hypothesis testing, as four predictor phenotypes were tested (the broad and narrow negative and disorganised syndromes) against a single outcome (narrow positive syndrome concordance). As the syndromes were correlated, an adjustment using the Bonferroni approach would be overconservative, so we used the Benjamini–Hochberg false discovery rate (FDR = 0.05).

## Results

### General characteristics of the samples

Descriptive statistics for the samples and proband characteristics are shown in Table 1 and Supplementary Tables 4–8, with further commentary in Supplementary Results.

### Concordance for psychotic syndromes

Concordances for the psychotic syndromes in the three schizophrenia samples combined (because of the limited sample size of the individual samples), the Maudsley register psychosis sample and Maudsley non-register psychosis samples are shown in Table 2. These were the groups for the main analysis but concordances for the three schizophrenia samples individually are also given in Supplementary Table 9 for further information.

**Table 2:** Probandwise concordance for positive, negative and disorganised psychotic syndromes in the five twin samples

Phenotypes		Samples					
Proband phenotype (present in all probands)	Co-twin phenotype (absent or present according to concordance)	Three schizophrenia samples combined (Fischer, Kringlen, Slater)		Maudsley register psychosis sample		Maudsley non-register psychosis sample	
		Concordance	% (95% CI)	Concordance	% (95% CI)	Concordance	% (95% CI)
Narrow positive syndrome (Pos = 2)	Narrow positive syndrome (Pos = 2)	Monozygotic 34/89	38.2 (28.1–48.3)	Monozygotic 29/70	41.4 (29.9–53.0)	Monozygotic 23/61	37.7 (25.5–49.9)
				Dizygotic 5/83	6.0 (0.91–11.1)	Dizygotic 1/16	6.3 (0.0–18.1)
Broad negative syndrome (Neg = 1 or 2)	Broad negative syndrome (Neg = 1 or 2)	Monozygotic 36/81	44.4 (33.6–55.3)	Monozygotic 16/52	30.8 (18.2–43.3)	Monozygotic 24/41	58.5 (43.5–73.6)
				Dizygotic 1/52	1.9 (0.0–5.7)	Dizygotic 1/10	10.0 (0.0–28.6)
Narrow negative syndrome (Neg = 2)	Narrow negative syndrome (Neg = 2)	Monozygotic 17/51	33.3 (20.4–46.3)	Monozygotic 2/12	16.7 (0.0–37.8)	Monozygotic 4/21	19.0 (2.3–35.8)
				Dizygotic 0/23	0.0	Dizygotic 0/2	0.0
Broad disorganised syndrome (Dis = 1 or 2)	Broad disorganised syndrome (Dis = 1 or 2)	Monozygotic 33/72	45.8 (34.3–57.3)	Monozygotic 32/57	56.1 (43.3–69.0)	Monozygotic 7/24	29.2 (11.0–47.4)
				Dizygotic 3/65	4.6 (0.0–9.7)	Dizygotic 0/7	0.0
Narrow disorganised syndrome (Dis = 2)	Narrow Disorganised syndrome (Dis = 2)	Monozygotic 6/31	19.4 (5.4–33.3)	Monozygotic 7/24	29.2 (11.0–47.4)	Monozygotic 2/5	40.0 (0.0–82.9)
				Dizygotic 0/27	0.0	Dizygotic 0/1	0.0

Pos, positive psychotic syndrome; Neg, negative psychotic syndrome; Dis, disorganised psychotic syndrome.

Narrow positive syndrome monozygotic concordances ranged from 37.7 to 41.4%, and 95% confidence intervals were all above zero, consistent with some familial influences. Confidence intervals were also above zero in each of the three schizophrenia samples individually. In the two psychosis samples, monozygotic concordances were greater than dizygotic concordances (monozygotic > dizygotic concordances) and 95% confidence intervals around the monozygotic and dizygotic concordances did not overlap, which was consistent with some genetic influences. In logistic regression analysis of the Maudsley register sample, monozygotic > dizygotic concordance was confirmed with adjustment for gender and age of co-twins at last information (odds ratio 9.27, 95% CI 2.97–28.91). In the Maudsley non-register sample, the association with zygosity had a similar odds ratio (9.77; 95% CI 0.97–98.83), but was not significant (95% confidence interval overlapped 1) in this smaller sample (Supplementary Table 10).

Results for the broad negative and disorganised syndromes were similarly consistent with some familial and genetic influences (monozygotic concordances significantly >0; and monozygotic >dizygotic concordances in samples with concordant dizygotic pairs). Results for the narrow negative and disorganised syndromes were consistent with some familial influences, but there were no concordant dizygotic pairs in the psychosis samples to allow calculation of confidence intervals around concordances and logistic regression odds ratios, possibly because the narrow syndromes were less common than the broad syndromes (see Supplementary Results for details).

### Heritability of psychotic syndromes

Twin modelling results for the psychotic syndromes in the Maudsley register psychosis sample are shown in Table 3. The twin probandwise concordances and lifetime morbid risks were the foundation for calculating the tetrachoric correlations and ACE model parameter estimates including heritability (*a* ^2^ or *h* ^2^). For all of the positive, negative and disorganised syndromes, the tetrachoric correlation was greater for monozygotic than dizygotic pairs, consistent with some genetic influences, although there was overlap in the 95% confidence intervals for the narrow negative syndrome. In the ACE models, all syndromes showed significant heritability with *a* ^2^ point estimates between 0.61 and 0.90 (61–90%) and 95% confidence intervals above zero. Confidence intervals around heritability estimates overlapped so no syndrome clearly showed higher or lower heritability than others. The remaining variance in liability was attributed to individual-specific/non-shared environmental effects (*e* ^2^), as all syndromes had point estimates of zero for common/shared environmental effects (*c* ^2^).

**Table 3:** Probandwise concordances, tetrachoric correlations and twin model-fitting for the psychotic syndromes and narrow positive subsyndromes in the Maudsley register psychosis twin sample

Proband phenotype (present in all probands)	Co-twin phenotype (absent or present according to concordance)	Probandwise concordances (95% CI)		Lifetime morbid risk of phenotype (%)	Tetrachoric correlations (95% CI)		Twin modelling parameter estimates for ACE model (95% CI)		
		Monozygotic	Dizygotic		Monozygotic	Dizygotic	*a* ^2^	*c* ^2^	*e* ^2^
Main syndromes									
Pos = 2	Pos = 2	29/70 (41.4%)	5/83 (6.0%)	1.18	0.82 (0.73–0.89)	0.30 (0.10 to 0.47)	0.81 (0.58–0.88)	0.00 (0.00–0.00)	0.19 (0.12–0.29)
Neg = 1 or 2	Neg = 1 or 2	16/52 (30.8%)	1/52 (1.9%)	0.80	0.75 (0.63–0.85)	0.13 (−0.21 to 0.41)	0.73 (0.60–0.84)	0.00 (0.00–0.00)	0.27 (0.16–0.40)
Neg = 2	Neg = 2	2/12 (16.7%)	0/23 (0.0%)	0.27	0.66 (0.32–0.88)	−0.85 (not applicable to 0.50)	0.61 (0.15–0.85)	0.00 (0.00–0.33)	0.39 (0.15–0.73)
Dis = 1 or 2	Dis = 1 or 2	32/57 (56.1%)	3/65 (4.6%)	0.94	0.91 (0.84–0.96)	0.27 (0.03–0.48)	0.90 (0.83–0.95)	0.00 (0.00–0.00)	0.10 (0.05–0.17)
Dis = 2	Dis = 2	7/24 (29.2%)	0/27 (0.0%)	0.39	0.77 (0.59–0.90)	−0.87 (not applicable to 0.44)	0.75 (0.56–0.88)	0.00 (0.00–0.00)	0.25 (0.12–0.44)
Subsyndromes of Pos = 2									
Pos = 2 and Neg = 0	Pos = 2 and Neg = 0	7/28 (25.0%)	0/34 (0.0%)	0.48	0.73 (0.54–0.86)	−0.61 (not applicable to 0.38)	0.69 (0.31–0.84)	0.00 (0.00–0.32)	0.31 (0.16–0.50)
Pos = 2 and Neg = 1 or 2	Pos = 2 and Neg = 1 or 2	12/42 (28.6%)	1/49 (2.0%)	0.70	0.74 (0.59–0.85)	0.16 (−0.19 to 0.44)	0.72 (0.57–0.83)	0.00 (0.00–0.00)	0.28 (0.17–0.43)
Pos = 2 and Neg = 2	Pos = 2 and Neg = 2	2/11 (18.2%)	0/21 (0.0%)	0.25	0.68 (0.34–0.89)	−0.68 (not applicable to 0.53)	0.64 (0.00–0.87)	0.00 (0.00–0.56)	0.36 (0.13–0.70)
Pos = 2 and Dis = 0	Pos = 2 and Dis = 0	2/21 (9.5%)	2/28 (7.1%)	0.38	0.52 (0.20–0.75)	0.46 (0.15–0.69)	0.12 (0.00–0.74)	0.40 (0.00–0.65)	0.48 (0.25–0.72)
Pos = 2 and Dis = 1 or 2	Pos = 2 and Dis = 1 or 2	27/49 (55.1%)	3/55 (5.5%)	0.80	0.91 (0.83–0.96)	0.32 (0.08–0.53)	0.90 (0.66–0.95)	0.00 (0.00–0.00)	0.10 (0.05–0.17)
Pos = 2 and Dis = 2	Pos = 2 and Dis = 2	7/22 (31.8%)	0/24 (0.0%)	0.36	0.80 (0.62–0.91)	−0.65 (not applicable to 0.47)	0.78 (0.34–0.90)	0.00 (0.00–0.00)	0.22 (0.10–0.41)

ACE, model comprising additive genetic, common/shared environmental, and non-shared/individual-specific environmental effects; *a* ^2^ (or *h* ^2^), additive genetic effects (heritability); *c* ^2^, common/shared environmental effects; *e* ^2^, non-shared/individual-specific environmental effects; Pos, positive psychotic syndrome; Neg, negative psychotic syndrome; Dis, disorganised psychotic syndrome; not applicable indicates that the 95% CI lower limit was not given by Open Mx where there is a negative tetrachoric correlation due to no concordant pairs.

### Monozygotic concordance for narrow positive syndrome in absence or presence of other syndromes

Concordances are shown in Supplementary Table 11 and results of logistic regression analysis are given in Table 4.

**Table 4:** Logistic regression analysis – predicting probandwise concordance for the narrow positive psychotic syndrome (Pos = 2) in monozygotic twin pairs according to the absence or presence of negative or disorganised syndromes also occurring in probands^a^

Predictor phenotype in probands	Samples								
	Three schizophrenia samples combined (Fischer, Kringlen, Slater)			Maudsley register psychosis sample			Maudsley non-register psychosis sample		
	Number of probandwise monozygotic pairs	Odds ratio (95% CI)	*p*-value	Number of probandwise monozygotic pairs	Odds ratio (95% CI)	*p*-value	Number of probandwise monozygotic pairs	Odds ratio (95% CI)	*p*-value
Pos = 2 and:									
Broad negative syndrome (Neg = 0 *v*. Neg = 1 or 2)	89	2.20 (0.71–6.82)	0.169	70	1.12 (0.37–3.33)	0.841	61	0.84 (0.22–3.25)	0.799
Narrow negative syndrome (Neg = 0 or 1 *v*. Neg = 2)	89	2.09 (0.68–6.47)	0.198	70	1.20 (0.27–5.21)	0.810	61	1.71 (0.46–6.40)	0.420
Broad disorganised syndrome (Dis = 0 *v*. Dis = 1 or 2)	89	3.47 (1.04–11.54)	0.043^*^	70	7.68 (1.49–39.70)	0.016^*^	61	3.48 (0.87–13.93)	0.076
Narrow disorganised syndrome (Dis = 0 or 1 *v*. Dis = 2)	89	2.17 (0.62–7.51)	0.220	70	1.78 (0.57–5.61)	0.320	61	0.81 (0.05–12.43)	0.874

Pos, positive psychotic syndrome; Neg, negative psychotic syndrome; Dis, disorganised psychotic syndrome.

An odds ratio >1 indicates higher narrow positive psychotic syndrome concordance when negative or disorganised syndrome also occurs in probands.

a. Generalised linear mixed model: absence or presence of the narrow positive psychotic syndrome in co-twin as dependent variable, adjusted for gender and age of co-twin at last information. Additionally, in analysis of the Maudsley register sample, twin pair modelled as a random effect to account for doubly ascertained pairs and a triplet proband with two co-twins. And in the three schizophrenia samples combined, sample and twin pair modelled as random effects.

* *p* < 0.05, two-tailed (nominally statistically significant, but not after adjustment for testing four correlated phenotypes using the Benjamini–Hochberg false discovery rate (FDR = 0.05)).

Among monozygotic pairs where probands had the narrow positive syndrome, concordance for the narrow positive syndrome was lower in the absence of (and higher in the presence of) the broad disorganised syndrome also occurring in probands, in the schizophrenia samples combined and the Maudsley register psychosis sample, including with adjustment for gender and age of co-twin at last information in logistic regression analysis (in the three schizophrenia samples: 89 pairs, odds ratio 3.47 (95% CI 1.04–11.54), *p* = 0.043; and the Maudsley register psychosis sample: 70 pairs, odds ratio 7.68 (95% CI 1.49–39.70), *p* = 0.016). This is consistent with a lower level of familial influences on the narrow positive syndrome when it occurs without disorganised symptoms, and *vice versa*. An important caveat is that, while the association with disorganised symptoms was found to be nominally statistically significant in both the schizophrenia samples and the Maudsley register sample independently, the individual results did not remain significant after adjustment for testing four phenotypes using the Benjamini–Hochberg FDR (FDR = 0.05). In the Maudsley non-register sample, there was a trend in the same direction, but the result was non-significant (95% confidence interval overlapped 1).

Negative symptoms were not significantly associated with concordance. However, in the schizophrenia samples, there was a modest non-significant trend toward higher concordance in the presence of negative symptoms (odds ratios of around 2.0), so the possibility of a scientifically meaningful association of small effect with negative symptoms in some samples is not completely excluded.

### Concordance for narrow positive subsyndromes

These results are shown in Supplementary Table 12.

Monozygotic concordance for the narrow positive syndrome with no disorganised symptoms (Pos = 2 and Dis = 0) was lower than for the narrow positive syndrome occurring with the broad disorganised syndrome (Pos = 2 and Dis = 1 or 2) (non-overlapping 95% confidence intervals) in the Maudsley register sample (concordance 9.5 *v*. 55.1%).

### Heritability of narrow positive subsyndromes

Results for the narrow positive syndrome, subdivided according to whether negative or disorganised syndromes were also absent or present, are shown in Table 3.

Heritability estimates for the narrow positive syndrome were similar with or without co-occurrence of the broad or narrow negative syndrome. The heritability estimate for the narrow positive syndrome occurring with the narrow negative syndrome had a 95% confidence interval that included zero, probably because this syndrome combination was the least common of those investigated, as well as having the lowest point estimate of the subsyndromes involving negative symptoms (0.64 or 64%).

The heritability estimates for the narrow positive syndrome occurring with disorganised symptoms were significant, with point estimates of 0.90 (or 90%) and 0.78 (or 78%) for co-occurrence with the broad and narrow disorganised syndromes, respectively.

The heritability point estimate for the narrow positive syndrome in the absence of disorganised symptoms was lower (0.12 or 12%) and non-significant, as the 95% confidence interval included zero. However, the confidence interval overlapped with those for the narrow positive syndrome occurring with disorganised symptoms, so this relatively low heritability point estimate was not formally confirmed as being lower than the other heritability estimates. The monozygotic and dizygotic tetrachoric correlations for the narrow positive syndrome without disorganised symptoms both had 95% confidence intervals above zero, consistent with some familial influences.

### Sensitivity analysis: concordance for psychotic syndromes and subsyndromes in monozygotic twins with DSM-III-R/IV schizophrenia

The results for individual samples are shown in Supplementary Table 13, and results for combined samples are shown in Supplementary Table 14.

For the positive, negative and disorganised syndromes there were relatively consistent results across samples, and significant monozygotic concordances of 19.1–44.1% in the four systematically ascertained samples combined, and in all five samples combined, consistent with some familial influences, as in the main analysis above.

Regarding the narrow positive subsyndromes, the monozygotic concordance was significantly lower in the absence of disorganised symptoms (Pos = 2 and Dis = 0) (12.1%, 95% CI 1.0–23.3) than when the broad disorganised syndrome was also present (Pos = 2 and Dis = 1 or 2) (41.3%, 95% CI 30.5–52.0) (95% confidence intervals non-overlapping), in the four systematically-ascertained samples combined. However, the difference became non-significant in the five samples combined (19.4%, 95% CI 9.5–29.2 *v*. 34.7%, 95% CI 25.4–43.9). It is not clear if the addition of the Maudsley non-register sample results led to a more accurate overall result, or whether there was an issue related to non-systematic ascertainment or the relatively low frequency of disorganised symptoms in this sample.

In the Maudsley register sample, when confining to twins with DSM-III-R schizophrenia, the heritability estimate for the narrow positive syndrome (Pos = 2) remained similar (0.82, 95% CI 0.71–0.90), as did the narrow positive syndrome with disorganised symptoms (Pos = 2 and Dis = 1 or 2: 0.89, 95% CI 0.66–0.95). Heritability for the narrow positive syndrome without disorganised symptoms (Pos = 2 and Dis = 0) was technically zero because there were no concordant monozygotic pairs (0/8), but this is probably an underestimate because of the limited sample size.

## Discussion

### Narrow positive psychotic syndrome

The narrow positive psychotic syndrome, comprising delusions and hallucinations co-occurring on a lifetime basis, showed evidence of familial and genetic influences across the twin samples and significant heritability in the Maudsley register sample (*h* ^2^ of approximately 80%), with the remaining variance in liability attributed to individual-specific/non-shared environmental influences. This is similar to estimates of twin heritability and environmental influences for operationally defined schizophrenia^1,2,6^ and schizoaffective disorder,^1^ where delusions and hallucinations are the commonest co-occurring core symptoms. However, these general results may hide variation in the strength of different aetiological influences, depending on the context in which the positive symptoms occur. In the current study there is provisional evidence of relatively low familial influences when the narrow positive syndrome occurred in the absence of disorganised symptoms, and relatively high familial influences when disorganised symptoms were also present. These familial influences are probably mainly genetic, in view of the absence of notable common/shared environmental influences in the twin modelling. An important caveat is that, although there was a nominal association with disorganised symptoms in two independent analyses, the individual associations did not withstand adjustment for multiple statistical testing. However, the results are consistent with studies of specific genetic and environmental risk factors, as outlined below.

Consistent with the narrow positive syndrome in isolation having a relatively high weighting of environmental risk factors, in the two psychosis twin samples investigated here along with other general clinical samples of individuals with psychotic disorders, we have previously found that co-occurring delusions and hallucinations have relatively distinct associations with predominantly environmental factors such as ethnic minority status and cannabis use, independently of negative and disorganised symptoms.^17^ Others have also found relatively distinct associations between positive symptoms and these factors,^15,20,21^ and for childhood adversity,^18^ including for co-occurring delusions and hallucinations.^19^

Consistent with the narrow positive syndrome in isolation having a corresponding relatively low weighting of genetic risk factors, among individuals with schizophrenia, higher levels of positive symptoms are usually not associated with higher familial or genetic loading for schizophrenia/psychosis.^24^ Additionally, there is evidence from adoption study data that higher levels of positive symptoms are associated with lower risk of schizophrenia in biological relatives of adoptees with schizophrenia (i.e. lower genetic loading);^39^ and in studies of polygenic risk score for schizophrenia, a measure of genetic loading due to commonly occurring risk variants, evidence of relatively low genetic loading among individuals with schizophrenia in the absence of disorganised symptoms^14,16,25^ and also sometimes in the absence of negative symptoms.^24,25^ And conversely, schizophrenia polygenic risk score is often higher when – in addition to positive symptoms – disorganised and/or negative symptoms are also present (see below).

The syndrome of delusions plus hallucinations probably also has relatively high genetic influences in at least some instances where it co-occurs with severe affective disorders. Of particular relevance, in schizoaffective disorders, delusions and hallucinations commonly co-occur, there is high twin heritability^1^ and elevated genetic loading for both schizophrenia and affective disorders.^40,41^

Studies based on relatively broad ranges of psychotic disorders show mixed results, as previously discussed,^42^ with some finding associations between higher levels of positive symptoms and schizophrenia polygenic risk score, and others not. The reasons for the differing results are not clear, and considerable methodological heterogeneity makes the studies difficult to compare.

Finally, research into milder psychotic experiences in the general population is consistent with the current study findings. High scores on paranoia plus hallucinations has relatively low heritability in adolescent twins (approximately 17%),^43^ and is usually not associated with higher schizophrenia polygenic risk score,^44^ but is associated with environmental factors such as bullying and cannabis use.^43^

In summary, when delusions and hallucinations occur in relative isolation from other major mental health symptoms, they may be predominantly influenced by environmental factors, with genetic factors becoming more pronounced when they occur in the lifetime context of disorganised symptoms, and sometimes negative symptoms or severe affective disorders.

### Negative psychotic syndromes

In the current study, the negative psychotic syndromes showed evidence of familial and genetic influences across the twin samples. Some investigations of genetic effects were not feasible for the narrow syndrome, as there were no concordant dizygotic pairs for this relatively uncommon phenotype. However, both broad and narrow syndromes showed evidence of significant heritability in the Maudsley register sample (*h* ^2^ of around 60–70%).

Monozygotic concordance and heritability of the narrow positive syndrome did not formally differ according to the absence or presence of negative symptoms. However, there was a trend towards higher monozygotic concordance in the presence of negative symptoms in the schizophrenia samples (odds ratios of around 2), so a meaningful association in some samples is not excluded.

In other studies results are variable. Among probands with schizophrenia, higher levels of negative symptoms are associated with higher familial risk of schizophrenia/psychosis,^24^ with evidence of higher genetic loading from a schizophrenia adoption study.^39^ Likewise, higher levels of negative symptoms in individuals with schizophrenia are associated with higher schizophrenia polygenic risk score.^24,25^ However, negative and disorganised symptoms are usually correlated, as in the current study samples, and when some researchers have tried to disaggregate them^14^ or investigated the independence of their associations,^16^ they have found the association to be predominantly with the disorganised, rather than negative, symptoms.

Investigations of negative symptoms are also complicated by the possibility of a mixture of symptoms due to primary developmental processes, and secondary to, for example, positive symptoms, medication side-effects or social isolation. The relative balance of primary and secondary negative symptoms may account for some of the differing results across samples, but clarifying this issue is difficult because of the difficulty distinguishing between primary and secondary negative symptoms in clinical samples.

In summary, there is evidence of genetic influences on negative psychotic symptoms, but the extent to which these are independent of influences on disorganised symptoms is not clear, nor is the extent to which the balance of primary and secondary negative symptoms causes variation in results across samples.

### Disorganised psychotic syndromes

In the current study, the disorganised psychotic syndromes showed evidence of familial and genetic influences across the twin samples. As with the negative syndromes, some investigations of genetic effects were not feasible for the narrow syndrome, as there were no concordant dizygotic pairs for this relatively uncommon phenotype. However, both broad and narrow syndromes showed evidence of significant heritability in the Maudsley register sample (*h* ^2^ of around 75–90%).

The narrow positive syndrome had nominally higher monozygotic concordance, and a trend toward a higher heritability estimate, in the presence versus absence of disorganised symptoms. Consistent with these findings, in other studies higher levels of disorganised symptoms are associated with higher schizophrenia polygenic risk score.^14,16,25^

There is also evidence of some overlap in familial influences on premorbid IQ and disorganised symptoms,^17^ so some genetic influences on disorganised symptoms may act via early life effects on cognition, or have pleiotropic effects on both cognition and symptoms. As well as common genetic and environmental factors influencing intelligence, premorbid IQ in schizophrenia is associated with chromosomal copy number variants and rare coding risk variants for schizophrenia,^45^ but the extent to which they influence disorganised symptoms is not yet known.

It is also possible that there are notable differences in aetiology of the disorganised syndrome according to context, e.g. potentially from the level of co-occurring cognitive impairment.

### Strengths and limitations

To our knowledge, this is the first study to investigate familial and genetic effects on positive, negative and disorganised psychotic syndromes in multiple schizophrenia and psychosis twin samples. Four of the five samples were systematically ascertained, and all of these had long follow-up and detailed clinical information from personal interviews and case records.

The systematically ascertained samples were ‘old’ (years of birth 1878–1986), but this coincided with an era of detailed clinical note-taking. In the schizophrenia samples, treatment with antipsychotic medication was often absent or occurred late in people’s histories, so there was potentially less misclassification of, for example, medication side-effects as negative symptoms. These samples also coincided with an era of institutionalisation, and recruitment did specifically include from among long-term mental hospital residents in the Slater study, so some negative symptoms could have been secondary to institutionalisation. On the other hand, in the Scandinavian samples, individuals often lived in private houses or on farms with paid or family carers.

Results of more recent studies of measured environmental and molecular genetic risk factors have been broadly consistent with results of the current study, but most research has involved participants of European ancestry. There is now a range of initiatives to increase the global diversity of research participants, including by the Psychiatric Genomics Consortium (https://pgc.unc.edu).

Possible changes to the pattern of results over time is necessarily speculative. However, since the samples were ascertained, there has been a marked increase in the frequency and potency of cannabis use. To the extent that cannabis is predominantly an environmental risk factor for positive symptoms, this could potentially lead to a decrease in the heritability of psychotic syndromes that exclusively or predominantly feature positive symptoms.

There has also been a marked increase in the use of antipsychotic medication and psychosocial treatments, including in the context of early intervention services. These developments have probably mainly attenuated the severity of positive symptoms, with possibly some additional attenuation of secondary negative symptoms, but probably not affected the basic pattern of relationships between symptoms and genetic and environmental risk factors indicated by the current twin study and other relevant studies of specific measured risk factors.

Information on symptoms was only systematically available for dizygotic pairs in the two psychosis samples, but the patterns of monozygotic concordance could be compared and contrasted across all five samples.

Although some twin-specific effects cannot be excluded, the rates of psychotic disorders are similar in twins and singletons,^1^ and among individuals with psychosis, the patterns of association with demographic, developmental and clinical variables are similar between twin and general clinical samples.^17^ Heritability for psychotic disorders is higher in twin studies (*h* ^2^ of around 80%)^1^ than in studies of other family/adoption relationships (*h* ^2^ of around 60%).^46^ The reasons for this difference are not clear, but may be partly attributable to only monozygotic twins sharing factors such as *de novo* genetic variants that are present before the twins divide.

Some assumptions of twin studies are not feasible to fully investigate, notably that twins in monozygotic and dizygotic pairs share common environmental risk factors to an equal extent, including *in utero*.^1,23^ However, the main findings of previous psychosis twin studies, such as substantial heritability of the main psychotic syndromes based on operational diagnoses^1^ and partial overlap in genetic liability between psychotic syndromes,^40^ are consistent with the results of molecular genetic investigations to date.^9^

To summarise, there are probably many pathways leading to the syndrome of delusions plus hallucinations. Taking into consideration co-occurrence with other psychotic symptoms, as well as severe affective disorders, may give valuable insights into the varying aetiologies underlying psychotic syndromes. In this context, the findings of the current study may be further evaluated in ongoing molecular genetic and environmental risk factor research.

## Supporting information

Cardno et al. supplementary material 1 Cardno et al. supplementary material

Cardno et al. supplementary material 2 Cardno et al. supplementary material

## Data Availability

The case histories from the Fischer, Kringlen and Slater samples have been published.^26–29^ OPCRIT variable definitions and additional guidance, originally written by L.A. Jones, A.G.C. and colleagues for a previous symptom dimensions study, are available on reasonable request to A.G.C. The raw numbers of total and concordant twin pairs for each syndrome, on which the concordances were based, are given in the main and supplementary results tables. The Open Mx R script for calculating the tetrachoric correlations and twin modelling was written by F.V.R. and is available at: https://ibg.colorado.edu/cdrom2016/rijsdijk/ThresholdLiabilityModels/. Any requests for other data sharing should be directed to A.G.C.

## Supplementary material

The supplementary material is available online at https://doi.org/10.1192/bjo.2026.11017
