# Supplementary material for: Concordance of positive, negative and disorganised psychotic syndromes in five twin samples
Source: BJPsych Open. 2026 Apr 1;12(3):e98. doi: 10.1192/bjo.2026.11017 (PMC13106995; doi:10.1192/bjo.2026.11017)
Supplement: Cardno et al. supplementary material 1 — Cardno et al. supplementary material [file S2056472426110175sup001.docx]

**Concordance of positive, negative and disorganised psychotic syndromes in five twin samples**

Cardno AG, et al.

**Supplementary Method and Results**

| Index to Supplementary Method and Results | |
| --- | --- |
| Section | page |
|  |  |
| Supplementary method |  |
| Further information about the samples | 2 |
| Description of OPCRIT | 4 |
| Definitions of psychotic syndromes - further details | 4 |
| Rating process and inter-rater reliability of symptom dimensions | 5 |
| Rationale for psychotic symptom dimension definitions | 6 |
| Further information about symptom ratings | 6 |
| Comparison of the psychotic syndromes with diagnostic criteria for DSM schizophrenia | 7 |
| Twin concordance analysis methods - further details | 8 |
|  |  |
| Supplementary results |  |
| General characteristics of the samples - further details | 12 |
| Concordance for negative and disorganised syndromes - further details | 14 |
|  |  |
| References | 15 |

**Supplementary method**

Further information about the samples

The Fischer schizophrenia sample^1^ was systematically ascertained by Margit Fischer. Twin probands were identified from the Danish twin register, cross-checked with the Danish central psychiatric register based on mental hospital admissions. Zygosity was assessed using blood markers and physical similarity questionnaires. Published case histories were available for monozygotic (MZ) pairs where probands had clinical schizophrenia, as assessed by the author, based on personal interviews and review of case records.

The Kringlen schizophrenia sample^2^ was systematically ascertained by Einar Kringlen, as part of a broader study of functional psychoses in twins. Twin probands were identified from the Norwegian birth register, cross-checked with the Norwegian central register of psychosis based on mental hospital admissions. Zygosity was assessed by blood markers and physical similarity questions. Published case histories^3^ were available for MZ pairs where probands had clinical schizophrenia or schizophreniform disorder, as assessed by the author, based on personal interviews and review of case records.

(A further Nordic twin study with published case histories by Tienari^4^ in Finland was considered, but excluded because there were no concordant MZ pairs at the time of publication and, although some co-twins became ill on further follow-up,^5^ published case histories relating to them are not available to our knowledge.)

The Slater schizophrenia sample^6^ was systematically ascertained by Eliot Slater, with the assistance of James Shields, as part of a broader study of psychotic and neurotic illnesses in twins. Recruitment of twin probands aimed to be unselected and comprehensive from the standing population of 10 mental hospitals for adult psychotic patients in and around London, UK; new admissions to these hospitals in 1936-39; and new inpatient or outpatient admissions to the Maudsley Hospital, London, in 1936-39. Zygosity was assessed by examination of physical similarity, fingerprints and personal history of similarity. Published case histories were available for MZ pairs where probands had clinical schizophrenia, as assessed by the author, based on personal interviews and review of case records. One MZ twin pair was also included in the Maudsley register psychosis sample (see below) and was excluded from analysis of the Slater sample in the current study to avoid overlap between samples. There were published case histories for some DZ pairs but these were not included in the current study as they were only for selected pairs.

Initial and final numbers for the current study derived from the Fischer, Kringlen and Slater samples are shown in Supplementary Table S1.

The Maudsley register psychosis twin sample^7^ was systematically ascertained from the Maudsley Twin Register, which was established by Eliot Slater. Probands comprised all individuals of multiple birth who attended any facility of the Maudsley and Bethlem Royal Hospitals in London between 1948 and 1993, and had psychotic symptoms or a manic/hypomanic episode. The twins with clinical schizophrenia investigated by Gottesman and Shields^8^ were included in the sample with additional follow-up. There were 223 probands and 224 probandwise pairs – the additional pair was from a triplet proband paired with their two co-triplets. 14 second onset co-twins with psychosis were independently ascertained as probands (28 doubly-ascertained pairs). Study assessments were based on clinical research interviews^8,9^ and review of case records by research psychiatrists or psychologists.

The Maudsley non-register psychosis twin sample^10,11^ was based on the combined Maudsley schizophrenia and bipolar twin study samples. Probands with schizophrenia or bipolar disorder and their co-twins were recruited nationally through the UK National Health Service by referrals from treating psychiatrists and through voluntary support groups. It was not systematically ascertained. Individuals with schizophrenia who had harmful substance use or dependence in the past 12 months were excluded. There were 123 probands, 112 of whom had a recruited co-twin. No pairs were doubly-ascertained. Study assessments were based on clinical research interviews^9,12,13^ and review of case records by research psychiatrists or psychologists.

In both twin psychosis samples, zygosity was assessed by physical similarity questionnaires and genetic markers.^7,10,11^

The samples have been employed previously for research based on operational diagnoses^7,10,11,14,15^ and case-only investigations of symptom dimensions^16-19^, but not for research into positive, negative and disorganised psychotic syndromes in their own right.

Description of OPCRIT

The Operational Criteria Checklist (OPCRIT)^20^ is a checklist containing 90 items, for rating psychotic and affective symptoms (we used binary (present/absent) ratings for the current study) along with risk factors and illness history variables. It was designed to be rated from multiple sources including lifetime clinical research interviews and review of case records. OPCRIT has established reliability^20,21^ and is a convenient and widely used assessment tool. OPCRIT is agnostic regarding symptom dimensions, i.e. it was not created with particular symptom dimensions in mind.

In the current study, lifetime ratings were made based on all relevant information from research interviews and clinical case records, or from case histories based on interviews and case record reviews.

In addition to the definitions in OPCRIT, raters used an OPCRIT raters' handbook with further guidance based on fieldwork for other schizophrenia symptom dimensions research^22^ (available on reasonable request to AGC).

Definitions of psychotic syndromes - further details

Psychotic syndromes were derived from the following psychotic symptom dimensions (dimensions originally scored 0-2):

Positive dimension (Pos) (scored 0-2): delusions/thought interference (score 1) and hallucinations (score 1).

Delusions/thought interference rated as present (score 1) if any of the OPCRIT items 54, 55, 57-71 rated as present.

Hallucinations rated as present (score 1) if any of the OPCRIT items 64, 65, 72-77 rated as present.

Narrow positive syndrome defined as present if Pos = 2.

Negative dimension (Neg) (scored 0-2): restricted/blunted affect (score 1) and negative formal thought disorder (i.e. poverty of speech) (score 1).

Restricted/blunted affect rated as present (score 1) if either OPCRIT item 32 or 33 rated as present.

Negative formal thought disorder rated as present (score 1) if OPCRIT item 29 rated as present.

Broad negative syndrome defined as present if Neg = 1 or 2.

Narrow negative syndrome defined as present if Neg = 2.

Disorganised dimension (Dis) (scored 0-2): positive formal thought disorder (score 1) and inappropriate affect (score 1).

Positive formal thought disorder rated as present (score 1) if either OPCRIT item 27 or 28 rated as present.

Inappropriate affect rated as present (score 1) if OPCRIT item 34 rated as present.

Broad disorganised syndrome defined as present if Dis = 1 or 2.

Narrow disorganised syndrome defined as present if Dis = 2.

Rating process and inter-rater reliability of symptom dimensions

In the schizophrenia samples, AGC made all OPCRIT ratings from the published case histories.

In the Maudsley register psychosis sample, inter-rater reliability (mean r_s_ between raters for 0-2 scored symptom dimensions in 30 cases) between the research psychiatrists (including AGC) and research psychologist who made ratings for the register twins was: Pos 0.69; Neg 0.65; Dis 0.66. Mean kappa between raters for DSM-III-R schizophrenia diagnosis was 0.73.

In the Maudsley non-register sample, ratings were made by research psychiatrists (including EV). This did not include formal assessment of inter-rater reliability but the raters frequently discussed cases and consulted with AGC.

Rationale for psychotic symptom dimension definitions

Psychotic symptom dimensions, from which the psychotic syndromes were derived, can also be based on, e.g. factor scores, which could retain more information and power. However, we found in previous analyses of familial aggregation in twins^16^ and siblings^22^ that the above OPCRIT symptom score definitions performed as well as OPCRIT or SAPS/SANS^23,24^ factor scores or more complex symptom scores, and are simpler to interpret and to convert to psychotic syndromes.

Pos can have subdivisions but global delusions and hallucinations are the most consistent core symptoms for the positive dimension in factor analyses.^25-28^ The original studies have most commonly been based on SAPS/SANS global symptoms but we have translated the factor solutions into 0-2 OPCRIT symptom dimension scores and found similar levels of familial aggregation, as described above.^22^ Neg can include symptoms related to reduced motivation/socialisation,^29^ but these are not available in OPCRIT and can be difficult to rate on a lifetime basis from interviews and case records. Dis can include bizarre behaviour, but we found in analysis of another sample that the confirmatory factor model fit was less good when this symptom was included.^29^

Factor analyses are also often based on PANSS,^30^ but we did not focus on these factor solutions as restricted/blunted and inappropriate affect are rated on the same variable, so negative and disorganised dimensions are less clearly separated.

We did not include affective symptoms in the analyses as these were assessed in different ways in different samples, and the concordances and heritability of mania, depressive psychosis, schizoaffective-mania and schizoaffective-depression syndromes have been investigated in the Maudsley register sample.^7,31^ We have also found that higher scores on each of the psychotic symptom dimensions are associated with lower overall prominence of affective symptoms in the two psychosis twin samples, as well as in general clinical psychosis samples.^17^

Further information about symptom ratings

In the current study we wanted to rate symptoms, and syndromes, as simply present or absent, based on all relevant information from research interviews and clinical case records.

If there was evidence of a symptom, it was rated as present. If there was no evidence of a symptom, despite research interviewers and clinicians aiming to elicit them, or if information regarding a symptom was ambiguous, it was rated as absent.

An example of an ambiguous description is when a patient was observed to be murmuring to themselves, and clinical staff wondered whether they might be hallucinating, but it was not confirmed that they were hearing voices.

In a previous study that investigated correlations of psychotic symptom dimensions in pairs of siblings affected by schizophrenia,^22^ we found that ratings based on SAPS/SANS, which includes the option of rating ambiguous descriptions as uncertain, did not give notably different within-pair correlations compared with rating symptoms as present/absent, as above.

Where there was uncertainty about other variables, e.g. the order of birth within a twin pair, this was rated as missing and omitted from the analysis, with the sample size used for analysis noted in the results.

Comparison of the psychotic syndromes with diagnostic criteria for DSM schizophrenia

The core symptoms on which the psychotic syndromes were based include most of the core symptoms for DSM schizophrenia (including DSM-5-TR), i.e. delusions, hallucinations, disorganized speech, and negative symptoms of diminished emotional expression.

The syndromes do not include grossly disorganized or catatonic behavior, nor negative symptoms of avolition. However, retarded catatonia, e.g. stupor, was rated under restricted affect and poverty of speech, i.e. negative symptoms of diminished emotional expression.

Inappropriate affect is not a core diagnostic symptom of DSM schizophrenia but is recognised to occur as part of the disorder.

For the main syndrome analysis, we did not apply the duration criteria for symptoms or reduced level of functioning, nor the relative balance of psychotic and affective symptoms.

We rated symptoms as present unless the research interview or clinical record clearly stated that they were caused by the physiological effects of a substance or another medical condition.

We rated delusions and hallucinations as present if they occurred in the context of an autism spectrum disorder or communication disorder of childhood onset.

For the sensitivity analysis, we rated symptoms as present only if the criteria for a main-lifetime diagnosis of DSM-III-R/IV schizophrenia were also fulfilled.

Twin concordance analysis methods - further details

*Concordance for psychotic syndromes*

An overview of the twin concordance analysis plan is given in Supplementary Table S3.

In each sample, we tabulated the probandwise concordance for each psychotic syndrome in MZ and also DZ twin pairs, where applicable, i.e. the lifetime prevalence of the syndrome in co-twins of probands with the syndrome. We also tabulated the pooled MZ concordances across the three schizophrenia samples - we used this for main analysis as sample sizes were modest for each individual schizophrenia sample - but also gave descriptive results for each individual sample in a supplementary file for further information. We calculated 95% confidence intervals (95%CIs) around concordances. We regarded MZ concordances with 95%CIs which did not include zero as consistent with some familial influences on the syndrome, and MZ > DZ concordances with non-overlapping 95%CIs as consistent with some genetic influences.

In the two psychosis samples, which had both MZ and DZ pairs, we conducted logistic regression analysis in a generalized linear mixed model framework in pairs where probands had the psychotic syndrome, with absence/presence of the syndrome in co-twins as the dependent variable and zygosity as the independent variable, adjusted for sex and age of co-twin at last information, and with twin pair modelled as a random effect to account for doubly-ascertained pairs in the Maudsley register sample. We regarded a significant effect of zygosity (MZ > DZ and 95%CI of odds ratio (OR) not overlapping 1) as consistent with some genetic influences on the syndrome.

*Further information on the rationale for pooling the schizophrenia samples*

We pooled the three schizophrenia samples for the main analyses as we were concerned that the sizes of individual samples might be too small for meaningful analysis, especially for subdivisions of the main syndromes, e.g. for the narrow positive syndrome occurring without negative symptoms.

We felt there was enough homogeneity between the samples to justify this, as all were systematically ascertained and assessed by experienced and dedicated psychiatrists, based on personal interviews and review of clinical records, who were employing northern European concepts of psychopathology in the first half of the 20th century. However, there was also some heterogeneity, e.g. relatively rural (Fischer, Kringlen) vs urban settings (Slater), breadth of inclusion criteria (schizophrenia only (Fischer, Slater) vs schizophrenia and schizophreniform psychosis (Kringlen)), and recruitment from long-stay wards in addition to incident recruitment in the Slater sample.

We gave the concordances for the main syndromes for each schizophrenia sample individually as additional supplementary information. And in logistic regression analysis of the three samples combined, within generalized linear mixed models, we modelled sample as a random effect in order to account for the clustering of pairs within samples.

*Suitability of the Maudsley register sample for twin modelling analysis*

The Maudsley register sample was suitable for twin modelling because it was a systematically-ascertained incident sample including both MZ and DZ pairs, and the lifetime morbid risk of syndromes could be estimated by extrapolation from local clinical case register and census data.^7^ The lifetime risk of syndromes and the probandwise MZ and DZ concordance data could then be input to Open Mx to calculate tetrachoric correlations and estimate heritability, shared and non-shared environmental effects in the ACE twin model, based on a maximum likelihood approach (https://openmx.ssri.psu.edu).

The Fischer and Kringlen samples were systematically-ascertained incident samples and lifetime risk of syndromes could theoretically be estimated from the national clinical register and population data, but there were no published case histories for DZ pairs so DZ concordance could not be assessed.

The Slater sample was systematically-ascertained, but included some pairs recruited from long-stay wards in addition to incident recruitment, and it was not clear which pairs were recruited in which way. Also there were published case histories for only selected DZ pairs so DZ concordance could not be assessed.

The Maudsley non-register sample included both MZ and DZ pairs, but was not systematically ascertained. In addition to making this sample unsuitable for twin modelling, the twin concordances needed to be treated with more caution than in the systematically-ascertained samples, but we were interested to include this sample for comparison with the systematically-ascertained samples.

*Heritability of psychotic syndromes*

In the Maudsley register sample, we calculated MZ and DZ tetrachoric correlations for each psychotic syndrome. These are based on the probandwise concordance and lifetime morbid risk of the phenotype within a liability-threshold model. For a given concordance, the tetrachoric correlation is higher when the phenotype is rarer, and vice versa. We regarded tetrachoric correlations where the 95%CI did not overlap zero as consistent with some familial influences, and MZ > DZ correlations with non-overlapping CIs as consistent with some genetic influences.

We estimated heritability within an ACE model, comprising additive genetic (a^2^ or h^2^), common/shared environmental (c^2^), and individual-specific/non-shared (e^2^) environmental influences; e^2^ also includes error variance due to, e.g. imperfect inter-rater reliability of the phenotype. Inputs are the MZ and DZ probandwise concordances, and lifetime morbid risk as a fixed effect. The model is based on the fact that MZ twins inherit virtually all of their segregating DNA sequence in common, while DZ twins inherit half of their segregating DNA sequence in common on average. In the ACE model, the MZ tetrachoric correlation (r) gives the upper limit of the possible heritability, which occurs when rMZ >= 2rDZ. (If rMZ > 2rDZ there may be non-additive/dominant genetic effects beyond the calculated additive genetic effects, but dominant (d^2^) effects were not modelled due to low power to detect these with the available sample size.) Where rMZ < 2rDZ this is consistent with some common environmental effects which maximize where rMZ = rDZ. Finally individual-specific effects reflect the extent to which rMZ is < 1.

*Further information about tetrachoric correlations*

Tetrachoric correlations are commonly used to assess correlations between two binary variables. A key advantage of this approach is that tetrachoric correlations are not sensitive to the frequencies of the variables in a population, so they can be used for common or uncommon variables within the limits of the sample size.

A key assumption is that the binary variables can be regarded in terms of a threshold on an underlying normal distribution, akin to dividing people according to whether they are tall or not tall on an underlying normal distribution of height.

For psychotic syndromes, the assumed underlying normal distribution is the liability to the syndrome, which is influenced by many genetic and environmental factors each of small effect. The fit of the liability model with one threshold cannot be formally tested, but it is consistent with current knowledge about the aetiology of psychotic disorders.

The position of the threshold on the normal liability distribution is determined by the lifetime morbid risk of the syndrome: if this is, e.g. 1%, then the threshold is positioned so that 1% of the normal distribution is to the right of this point. When complete population data is available, thresholds can be estimated from the data. For data from clinical case registers, all probands have the syndrome and the frequency of the syndrome in co-twins is known (giving the probandwise concordance). Thresholds cannot be estimated from the data because, e.g. the frequency of unaffected-unaffected twin pairs is not known, but tetrachoric correlations can still be calculated if the frequency of the syndrome in the population (i.e. the lifetime morbid risk) is known from other sources, e.g. derived from local clinical case register and census data, and the threshold is specified according to this information.

*MZ concordance for narrow positive syndrome in absence or presence of other syndromes*

In each sample we tablulated the MZ probandwise concordance for the commonest syndrome (the narrow positive syndrome) according to whether probands also had 0, 1 or 2 negative or disorganised symptoms. (There were insufficient concordant DZ pairs for DZ analysis.)

(We also considered investigating concordance for, e.g. the narrow negative syndrome in the absence or presence of disorganised symptoms, and vice versa, but the number of concordant pairs with these syndromes was too low to allow for this further sub-analysis to give meaningful results.)

We then conducted logistic regression analysis in a generalized linear mixed model framework in MZ pairs where probands had the psychotic syndrome, with absence/presence of the narrow positive syndrome in co-twins as the dependent variable and absence/presence of the broad or narrow negative or disorganised syndrome as the independent variable (as four separate analyses), adjusted for sex and age of co-twin at last information, and with twin pair modelled as a random effect to account for doubly-ascertained pairs. As the schizophrenia samples each had only modest numbers of pairs, we combined them in analysis and also modelled sample as a random effect. We regarded a significant effect of the independent variable (e.g. the broad negative syndrome) (with 95%CI of OR not overlapping 1) as consistent with a difference in concordance of the narrow positive syndrome according to the additional absence/presence of, e.g. negative symptoms, in probands.

We previously found in the Maudsley register sample, that adjustment for the small number of doubly-ascertained pairs was not required for twin modelling.^15^ The same probably applies for current study analyses of the Fischer and Slater samples, and is not applicable to the Maudsley non-register sample where there were no doubly-ascertained pairs. However, there was complete ascertainment in the Kringlen sample, meaning that both twins were ascertained in all concordant pairs. In view of this, modelling twin pair as a random effect in the logistic regression analyses was applicable in the Kringlen sample, and so we did the same in the other samples for consistency.

**Supplementary results**

General characteristics of the samples - further details

*Descriptive statistics for the samples*

These are shown in Table 1.

Compared with the systematically-ascertained Maudsley register sample, the non-systematically-ascertained Maudsley non-register sample had a lower proportion of DZ pairs.

The Slater schizophrenia sample had the lowest proportion of male probands. This might have been at least partly due to more young adult males than females being killed in the First World War.

All samples were predominantly or entirely of white ethnicity.

The Fischer schizophrenia sample had proband diagnoses most closely aligned with DSM-IV schizophrenia.

The broader psychosis samples had a lower proportion of probands with chronic illness course than the schizophrenia samples.

For the three schizophrenia samples, the year-of-birth range of probands overlapped, and in each case probands were older than in the Maudsley non-register sample, while the broader year-of-birth range of the Maudsley register sample overlapped with all of the other samples.

Mean age at onset and age of co-twin at last information was oldest in the Fischer schizophrenia sample and youngest in the Maudsley non-register twin sample.

Most probands had both delusions and hallucinations (Pos=2) and in most samples few had neither positive symptom (Pos=0). The frequencies of negative and disorganised symptoms were more evenly spread, although there were few probands with both formal thought disorder and inappropriate affect (Dis=2) in the Fischer and Maudsley non-register samples.

It might be expected that the Slater sample was most selected for severity, as it included recruitment from long-stay inpatient wards; and the Maudsley non-register sample least so, as most participants engaged in a range of cognitive and neuroimaging investigations. These two samples had the highest and lowest proportion of MZ probands with the narrow disorganised syndrome (Dis=2) (54.3% and 5.9%, respectively), which may reflect this.

*Correlations between psychotic symptom dimensions in probands*

Spearman correlations between psychotic symptom dimension 0-2 scores in probands are shown in Supplementary Table S4.

In the two psychosis samples, all psychotic symptom dimension scores were positively correlated. Correlations were generally lower in the schizophrenia samples, especially between the positive dimension and other dimensions. This may be due to the narrower ascertainment frame in the schizophrenia samples. Consistent with this, correlations in the Maudsley register sample reduced to levels similar to the schizophrenia samples in a post hoc sensitivity analysis confined to probands with DSM-III-R schizophrenia (Supplementary Table S5).

*Associations of psychotic syndromes with demographic, developmental and clinical variables in probands*

Descriptive statistics are shown in Supplementary Table S6 and results of logistic regression analysis in Supplementary Table S7.

For the purposes of description, results at p<0.01, two-tailed, are described as associated.

The negative syndrome was associated with male sex in the Maudsley non-register psychosis sample. All three syndromes were associated with younger age at onset in the Maudsley register psychosis sample. In the schizophrenia samples, the narrow negative and disorganised syndromes were also associated with younger age at onset, but the narrow positive syndrome was associated with older age at onset. Again this last finding may have been due to the narrower ascertainment frame in the schizophrenia samples. Consistent with this, associations between the narrow positive syndrome and age at onset in the two psychosis samples reduced to null (ORs ~1) in post hoc sensitivity analysis confined to probands with DSM-III-R/IV schizophrenia (Supplementary Table S8).

All three syndromes were associated with chronic illness course in the two psychosis samples, as was the broad and narrow disorganised syndrome in the schizophrenia samples, with trends in the same direction for the negative and positive syndromes.

Concordance for negative and disorganised syndromes - further details

These results are shown in Table 2 and Supplementary Tables S9 and S10.

Broad negative syndrome MZ concordances ranged from 30.8% to 58.5% and 95%CIs were all above zero. CIs were also above zero in the three schizophrenia samples individually. In the psychosis samples, there were MZ > DZ concordances with non-overlapping 95%CIs, and this was confirmed in logistic regression analysis with adjustment for sex and age of co-twin at last information.

Narrow negative syndrome MZ concordances ranged from 16.7% to 33.3%. 95%CIs were above zero except in the Maudsley register psychosis sample. CIs were above zero in the three schizophrenia samples individually. Neither psychosis sample had any concordant DZ pairs so 95%CIs in MZ and DZ pairs could not be compared, and logistic regression analysis of zygosity was not conducted.

Broad disorganised syndrome MZ concordances ranged from 29.2% to 56.1% and 95%CIs were all above zero. CIs were above zero in the Kringlen and Slater schizophrenia samples individually but not in the smaller Fischer sample. In the Maudsley register psychosis sample there was MZ > DZ concordance with non-overlapping 95%CIs, which was confirmed in logistic regression analysis; while the Maudsley non-register sample had no concordant DZ pairs for assessment.

Narrow disorganised syndrome MZ concordances ranged from 19.4% to 40.0%. 95%CIs were above zero except in the Maudsley non-register psychosis sample, where the narrow disorganised syndrome was relatively uncommon. The CI was above zero in the Slater schizophrenia sample on its own, but not in the Fischer nor Kringlen samples individually, where again the narrow disorganised syndrome was relatively uncommon. Neither psychosis sample had any concordant DZ pairs for comparison of MZ and DZ 95%CIs, or for logistic regression analysis of zygosity.

In summary, there was evidence consistent with some familial and genetic influences on the broad negative and disorganised syndromes. For the narrow negative and disorganised syndromes, there was evidence of familial influences but no concordant DZ pairs to allow calculation of CIs around concordances and logistic regression odds ratios, possibly because these syndromes were less common than the broad syndromes.

However, in the Maudsley register sample it was possible to investigate tetrachoric correlations in MZ and DZ pairs, and estimate heritability, for the broad and narrow negative and disorganised syndromes (see main text Results and Table 3).

**References**

1. Fischer M. Genetic and environmental factors in schizophrenia: a study of schizophrenic twins and their families. *Acta Psychiatr Scand* 1973; suppl 238.

2. Kringlen E. *Heredity and Environment in the Functional Psychoses: An Epidemiological-Clinical Twin Study.* London: William Heinemann, 1967.

3. Kringlen E. *Heredity and Environment in the Functional Psychoses: Case Histories.* Oslo: Universitetsforlaget, 1967.

4. Tienari P. Psychiatric illnesses in identical twins. *Acta Psychiatr Scand* 1963; **39** (Suppl. 171): 1-196.

5. Tienari P. Schizophrenia in Finnish male twins. In M. H. Lader (Ed.), Studies of schizophrenia. *Br J Psychiatry* 1975; **10**: 29-35.

6. Slater E. *Psychotic and Neurotic Illnesses in Twins.* London: HMSO, 1953.

7. Cardno AG, Marshall EJ, Coid B, Macdonald AM, Ribchester TR, Davies NJ, et al. Heritability estimates for psychotic disorders: the Maudsley twin psychosis series. *Arch Gen Psychiatry* 1999; **56**: 162-8.

8. Gottesman II, Shields J. *Schizophrenia and Genetics: A Twin Vantage Point.* Orlando, Fla: Academic Press Inc, 1972.

9. Spitzer RL, Endicott J. *Schedule for Affective Disorders and Schizophrenia: Lifetime Version.* New York: New York State Psychiatric Institute, 1978.

10. Toulopoulou T, Picchioni M, Rijsdijk F, Hua-Hall M, Ettinger U, Sham P, et al. Substantial genetic overlap between neurocognition and schizophrenia: genetic modeling in twin samples. *Arch Gen Psychiatry* 2007; **64**: 1348-55.

11. Georgiades A, Rijsdijk F, Kane F, Rebollo-Mesa I, Kalidindi S, Schulze KK, et al. New insights into the endophenotypic status of cognition in bipolar disorder: genetic modelling study of twins and siblings. *Br J Psychiatry* 2016; **208**: 539-47.

12. First MB, Spitzer RL, Gibbon M, Williams JBM. *Structured Clinical Interview for DSM-IV Axis I Disorders (SCID).* New York: New York State Psychiatric Institute, 1997.

13. Wing JK, Babor T, Brugha T, Burke J, Cooper JE, Giel R, et al. SCAN. Schedules for Clinical Assessment in Neuropsychiatry. *Arch Gen Psychiatry* 1990; **47**: 589-93.

14. Pepper EJ, Pathmanathan S, McIlrae S, Rehman FU, Cardno AG. Associations between risk factors for schizophrenia and concordance in four monozygotic twin samples. *Am J Med Genet Part B, Neuropsychiatr Genet* 2018; **177**: 503-10.

15. Cardno AG, Rijsdijk FV, Sham PC, Murray RM, McGuffin P. A twin study of genetic relationships between psychotic symptoms. *Am J Psychiatry* 2002; **159**: 539-45.

16. Cardno AG, Sham PC, Murray RM, McGuffin P. Twin study of symptom dimensions in psychoses. *Br J Psychiatry* 2001; **179**: 39-45.

17. Cardno AG, Allardyce J, Bakker SC, Toulopoulou T, Kravariti E, Picchioni MM, et al. Associations of psychotic symptom dimensions with clinical and developmental variables in twin and general clinical samples. *Br J Psychiatry* 2025; **226**: 16-23.

18. Rijsdijk FV, Gottesman II, McGuffin P, Cardno AG. Heritability estimates for psychotic symptom dimensions in twins with psychotic disorders. *Am J Med Genet Part B, Neuropsychiatr Genet* 2011; **156b**: 89-98.

19. Dworkin RH, Lenzenweger MF. Symptoms and the genetics of schizophrenia: implications for diagnosis. *Am J Psychiatry* 1984; **141**: 1541-46.

20. McGuffin P, Farmer A, Harvey I. A polydiagnostic application of operational criteria in studies of psychotic illness. Development and reliability of the OPCRIT system. *Arch Gen Psychiatry* 1991; **48**: 764-70.

21. Williams J, Farmer AE, Ackenheil M, Kaufmann CA, McGuffin P. A multicentre inter-rater reliability study using the OPCRIT computerized diagnostic system. *Psychol Med* 1996; **26**: 775-83.

22. Cardno AG, Jones LA, Murphy KC, Sanders RD, Asherson P, Owen MJ, et al. Dimensions of psychosis in affected sibling pairs. *Schizophr Bull* 1999; **25**: 841-50.

23. Andreasen NC. *The Scale for the Assessment of Negative Symptoms (SANS).* Iowa City, IA: University of Iowa, 1984.

24. Andreasen NC. *The Scale for the Assessment of Positive Symptoms (SAPS).* Iowa City, IA: University of Iowa, 1984.

25. Liddle PF. The symptoms of chronic schizophrenia. A re-examination of the positive-negative dichotomy. *Br J Psychiatry* 1987; **151**: 145-51.

26. Andreasen NC, Arndt S, Alliger R, Miller D, Flaum M. Symptoms of schizophrenia. Methods, meanings, and mechanisms. *Arch Gen Psychiatry* 1995; **52**: 352-60.

27. Grube BS, Bilder RM, Goldman RS. Meta-analysis of symptom factors in schizophrenia. *Schizophr Res* 1998; **31**: 113-20.

28. Dazzi F, Shafer A. Meta-analysis of the factor structure of the Scale for the Assessment of Negative Symptoms (SANS) and the Scale for the Assessment of Positive Symptoms (SAPS). *Schizophr Res* 2024; **274**: 464-72.

29. Legge SE, Cardno AG, Allardyce J, Dennison C, Hubbard L, Pardiñas AF, et al. Associations between schizophrenia polygenic liability, symptom dimensions, and cognitive ability in schizophrenia. *JAMA Psychiatry* 2021; **78**: 1143-51.

30. Kay SR, Fiszbein A, Opler LA. The positive and negative syndrome scale (PANSS) for schizophrenia. *Schizophr Bull* 1987; **13**: 261-76.

31. Cardno AG, Rijsdijk FV, West RM, Gottesman II, Craddock N, Murray RM, et al. A twin study of schizoaffective-mania, schizoaffective-depression, and other psychotic syndromes. *Am J Med Genet Part B, Neuropsychiatr Genet* 2012; **159b**:172-82.
