# Supplementary material for: Concordance of positive, negative and disorganised psychotic syndromes in five twin samples
Source: BJPsych Open. 2026 Apr 1;12(3):e98. doi: 10.1192/bjo.2026.11017 (PMC13106995; doi:10.1192/bjo.2026.11017)
Supplement: Cardno et al. supplementary material 2 — Cardno et al. supplementary material [file S2056472426110175sup002.docx]

**Concordance of positive, negative and disorganised psychotic syndromes in five twin samples**

Cardno AG, et al.

**Supplementary Tables**

| Index to Supplementary Tables | |
| --- | --- |
| Table No. | Title |
|  |  |
| Table S1 | Number of monozygotic (MZ) twin pairs in the Fischer, Kringlen and Slater schizophrenia twin samples |
| Table S2 | Definitions of positive, negative and disorganised psychotic syndromes |
| Table S3 | Twin concordance analysis plan |
| Table S4 | Spearman intercorrelations between psychotic symptom dimensions (0-2 scores) in probands in the five twin samples |
| Table S5 | Post hoc sensitivity analysis - Spearman intercorrelations between psychotic symptom dimensions (0-2 scores) in probands with DSM-III-R/IV schizophrenia in the five twin samples |
| Table S6 | Descriptive statistics of associations of demographic, developmental and clinical variables with psychotic syndromes in probands in the five twin samples |
| Table S7 | Logistic regression analysis of psychotic syndromes on demographic, developmental and clinical variables in probands in the five twin samples |
| Table S8 | Post hoc sensitivity analysis - Logistic regression analysis of the narrow positive psychotic syndrome on age at onset in probands with DSM-III-R/IV schizophrenia in the five twin samples |
| Table S9 | Probandwise concordance for positive, negative and disorganised psychotic syndromes in the three schizophrenia twin samples individually |
| Table S10 | Logistic regression analysis - zygosity as a predictor of concordance in the two psychosis twin samples |
| Table S11 | Probandwise concordance for the narrow positive psychotic syndrome (Pos=2) in MZ twin pairs according to the level of negative or disorganised symptoms also occurring in probands |
| Table S12 | Probandwise concordance for narrow positive sub-syndromes in the five twin samples |
| Table S13 | Sensitivity analysis - probandwise concordance for the psychotic syndromes and narrow positive sub-syndromes in MZ twins with DSM-III-R/IV schizophrenia in the five twin samples |
| Table S14 | Sensitivity analysis - probandwise concordance for the psychotic syndromes and narrow positive sub-syndromes in MZ twins with DSM-III-R/IV schizophrenia in the four systematically-ascertained samples combined, and in all five twin samples combined |
|  |  |

| Table S1. Number of monozygotic (MZ) twin pairs in the Fischer, Kringlen and Slater schizophrenia twin samples^a^ | | | |
| --- | --- | --- | --- |
| Number of MZ twin pairs | Samples | | |
|  | Fischer | Kringlen | Slater |
| Original number of pairs where at least one twin had schizophrenia as assessed by study author (also schizophreniform psychosis in Kringlen sample) | 21 | 55 | 41 |
| Number of excluded pairs^b^ | 0 | 6 | 10 |
| Number of pairs where co-twin also independently ascertained (doubly-ascertained pairs) | 4 | 17 | 4 |
| Number of probandwise pairs included in current study^c^ | 25 | 66 | 35 |
|  |  |  |  |
| ^a^For Fischer and Kringlen samples, case histories only available for MZ twins; for Slater sample, DZ case histories also available but only for selected pairs so DZ pairs not included in the current study.  ^b^Exclusions due to case history unavailable or insufficient symptom information (including where part of case history was in another publication unavailable for the current study), or no record of a lifetime positive, negative or disorganised psychotic symptom being present; additionally, one Slater pair excluded because also in Maudsley register sample.  ^c^Probands had at least one lifetime positive, negative or disorganised psychotic symptom recorded as present. | | | |

| Table S2. Definitions of positive, negative and disorganised psychotic syndromes | |
| --- | --- |
| Syndrome | Definition^a^ |
|  |  |
| Narrow positive (Pos=2) | Psychotic disorder with any delusions/thought interference and any hallucinations present |
|  |  |
| Broad negative (Neg=1or2) | Psychotic disorder with restricted affect and/or poverty of speech present |
| Narrow negative (Neg=2) | Psychotic disorder with restricted affect and poverty of speech present |
|  |  |
| Broad disorganised (Dis=1or2) | Psychotic disorder with positive formal thought disorder and/or inappropriate affect present |
| Narrow disorganised (Dis=2) | Psychotic disorder with positive formal thought disorder and inappropriate affect present |
|  |  |
| ^a^Lifetime-ever symptoms rated in OPCRIT | |

| Table S3. Twin concordance analysis plan | | | | | |
| --- | --- | --- | --- | --- | --- |
| Phenotype examples | | Analysis^a^ | | Criteria for statistical significance | Notes |
| Proband  [phenotype present in all probands] | Co-twin  [phenotype absent or present according to concordance] |  | |  |  |
| Probandwise concordance for main psychotic syndromes | |  | |  |  |
| e.g., Narrow positive (Pos=2) | Narrow positive (Pos=2) | Familial influences:  MZ concordance > 0%  [also DZ > 0% if sufficient concordant pairs] | | 95%CI not overlapping 0% | and non-familial influences if MZ concordance < 100% |
|  |  | Genetic influences:  MZ > DZ concordance  Logistic regression OR (for zygosity: MZ vs DZ) > 1 [generalized linear mixed model] | | 95%CIs of MZ and DZ concordances not overlapping  95%CI not overlapping 1 | Logistic regression analysis result takes precedence |
|  |  |  |  |  |  |
| Probandwise concordance for narrow positive syndrome in absence/presence of negative or disorganised syndromes also occurring in probands^b^ | |  |  |  |  |
| e.g., Pos=2 and Neg=0  compared with  Pos=2 and Neg=1or2 | Pos=2 | Familial influences:  Logistic regression OR (for Neg=1or2 vs Neg=0) > 1 [generalized linear mixed model] | | 95%CI not overlapping 1 | Insufficient concordant DZ pairs for DZ analysis |
|  |  |  |  |  |  |
| Probandwise concordance for narrow positive sub-syndromes | |  |  |  |  |
| e.g., Pos=2 and Neg=0 | Pos=2 and Neg=0 | Familial influences:  Concordances as for main psychotic syndromes | |  |  |
|  |  | Genetic influences:  Concordances as for main psychotic syndromes | |  | Logistic regression analysis omitted as most phenotypes had no concordant DZ pairs, so ORs not calculable |
|  |  |  |  |  |  |
| ^a^Genetic and environmental influences on main psychotic syndromes and narrow positive sub-syndromes also analysed with tetrachoric correlations and ACE twin models in Maudsley register sample.  ^b^Insufficient concordant pairs for narrow negative and disorganised syndromes for subdivisions of these to be analysed (e.g. concordance for narrow negative syndrome according to absence or presence of disorganised symptoms also occurring in probands). | | | | | |

| Table S4. Spearman intercorrelations between psychotic symptom dimensions (0-2 scores) in probands in the five twin samples | | | | |
| --- | --- | --- | --- | --- |
| Sample (no. probands) | Psychotic symptom dimensions (r_s_) | | | |
|  |  | Positive | Negative | Disorganised |
| Fischer schizophrenia  (n=25) | Positive | - | -0.26 | -0.30 |
|  | Negative |  | - | 0.11 |
|  |  |  |  |  |
| Kringlen schizophrenia  (n=66) | Positive | - | -0.14 | 0.05 |
|  | Negative |  | - | 0.21 |
|  |  |  |  |  |
| Slater schizophrenia  (n=35) | Positive | - | -0.13 | 0.09 |
|  | Negative |  | - | 0.37* |
|  |  |  |  |  |
| Maudsley register psychosis sample  (n=224) | Positive | - | 0.36*** | 0.40*** |
|  | Negative |  | - | 0.39*** |
|  |  |  |  |  |
| Maudsley non-register psychosis sample  (n=112) | Positive | - | 0.50*** | 0.26** |
|  | Negative |  | - | 0.23* |
|  |  |  |  |  |
| *p<0.05, **p<0.01, ***p<0.001, two-tailed. | | | | |

| Table S5. Post hoc sensitivity analysis - Spearman intercorrelations between psychotic symptom dimensions (0-2 scores) in probands with DSM-III-R/IV schizophrenia in the five twin samples | | | | |
| --- | --- | --- | --- | --- |
| Sample (no. probands) | Psychotic symptom dimensions (r_s_) | | | |
|  |  | Positive | Negative | Disorganised |
| Fischer schizophrenia  (n=24) | Positive | - | -0.17 | -0.48* |
|  | Negative |  | - | 0.18 |
|  |  |  |  |  |
| Kringlen schizophrenia  (n=40) | Positive | - | -0.18 | 0.03 |
|  | Negative |  | - | 0.22 |
|  |  |  |  |  |
| Slater schizophrenia  (n=23) | Positive | - | -0.20 | 0.02 |
|  | Negative |  | - | 0.25 |
|  |  |  |  |  |
| Maudsley register psychosis sample  (n=97) | Positive | - | 0.09 | -0.17 |
|  | Negative |  | - | 0.31** |
|  |  |  |  |  |
| Maudsley non-register psychosis sample  (n=68) | Positive | - | 0.30* | 0.15 |
|  | Negative |  | - | 0.02 |
|  |  |  |  |  |
| *p<0.05, **p<0.01, ***p<0.001, two-tailed. | | | | |

| Table S6. Descriptive statistics of associations of demographic, developmental and clinical variables with psychotic syndromes in probands in the five twin samples | | | | |
| --- | --- | --- | --- | --- |
| Demographic, developmental or clinical variable | Psychotic syndrome | Sample | | |
|  |  | Three schizophrenia samples combined (Fischer, Kringlen, Slater) | Maudsley register psychosis sample | Maudsley non-register psychosis sample |
|  |  |  |  |  |
| Sex (% male) | Pos=0 | 4/9 (44.4%) | 8/9 (88.9%) | 2/15 (13.3%) |
|  | Pos=1 | 15/28 (53.6%) | 32/62 (51.6%) | 9/20 (45.0%) |
|  | Pos=2 | 39/89 (43.8%) | 80/153 (52.3%%) | 44/77 (57.1%) |
|  |  |  |  |  |
|  | Neg=0 | 17/45 (37.8%) | 62/120 (51.7%) | 19/61 (31.1%) |
|  | Neg=1 | 13/30 (43.3%) | 39/69 (56.5%) | 18/28 (64.3%) |
|  | Neg=2 | 28/51 (54.9%) | 19/35 (54.3%) | 18/23 (78.3%) |
|  |  |  |  |  |
|  | Dis=0 | 25/54 (46.3%) | 53/102 (52.0%) | 40/81 (49.4%) |
|  | Dis=1 | 23/41 (56.1%) | 38/71 (53.5%) | 15/25 (60.0%) |
|  | Dis=2 | 10/31 (32.3%) | 29/51 (56.9%) | 0/6 (0.0%) |
|  |  |  |  |  |
| Birth order (% second born)  [includes one 3rd born triplet in Maudsley reg sample] | Pos=0 | 5/9 (55.6%) | 3/9 (33.3%) | No probands with birth order data and Pos=0 |
|  | Pos=1 | 14/27 (51.9%) | 30/50 (60.0%) | 4/5 (80.0%) |
|  | Pos=2 | 42/77 (54.5%) | 60/135 (44.4%) | 26/53 (49.1%) |
|  |  |  |  |  |
|  | Neg=0 | 23/39 (59.0%) | 51/99 (51.5%) | 12/17 (70.6%) |
|  | Neg=1 | 15/26 (57.7%) | 27/61 (44.3%) | 10/22 (45.5%) |
|  | Neg=2 | 23/48 (47.9%) | 15/34 (44.1%) | 8/19 (42.1%) |
|  |  |  |  |  |
|  | Dis=0 | 24/45 (53.3%) | 45/83 (54.2%) | 13/32 (40.6%) |
|  | Dis=1 | 22/39 (56.4%) | 24/63 (38.1%) | 13/21 (61.9%) |
|  | Dis=2 | 15/29 (51.7%) | 24/48 (50.0%) | 4/5 (80.0%) |
|  |  |  |  |  |
| Age at onset^a^ (years)  (n, mean, (sd)) | Pos=0 | 8  22.6 yrs (3.6) | 9  30.1 yrs (18.8) | 15  20.5 yrs (10.0) |
|  | Pos=1 | 27  26.7 yrs (9.6) | 62  30.8 yrs (13.0) | 19  20.5 yrs (5.1) |
|  | Pos=2 | 89  30.8 yrs (8.8) | 153  24.1 yrs (9.0) | 77  21.1 yrs (5.4) |
|  |  |  |  |  |
|  | Neg=0 | 44  32.0 yrs (9.1) | 120  28.1 yrs (12.3) | 61  20.3 yrs (6.9) |
|  | Neg=1 | 30  32.0 yrs (9.4) | 69  25.0 yrs (10.3) | 27  22.2 yrs (5.3) |
|  | Neg=2 | 50  25.4 yrs (7.2) | 35  22.1 yrs (5.9) | 23  21.3 yrs (4.4) |
|  |  |  |  |  |
|  | Dis=0 | 53  30.9 yrs (10.3) | 102  30.1 yrs (13.6) | 80  21.0 yrs (6.6) |
|  | Dis=1 | 40  30.4 yrs (7.6) | 71  23.2 yrs (7.1) | 25  20.9 yrs (4.4) |
|  | Dis=2 | 31  25.4 yrs (9.0) | 51  22.6 yrs (7.0) | 6  20.8 yrs (5.5) |
|  |  |  |  |  |
| Illness course (% chronic course) [from OPCRIT item 90 score 4 or 5] | Pos=0 | 3/7 (42.9%) | 1/7 (14.3%) | 0/9 (0.0%) |
|  | Pos=1 | 14/28 (50.0%) | 7/61 (11.5%) | 2/14 (14.3%) |
|  | Pos=2 | 55/88 (62.5%) | 60/151 (39.7%) | 33/65 (50.8%) |
|  |  |  |  |  |
|  | Neg=0 | 19/43 (44.2%) | 19/116 (16.4%) | 4/43 (9.3%) |
|  | Neg=1 | 18/30 (60.0%) | 27/68 (39.7%) | 14/26 (53.8%) |
|  | Neg=2 | 35/50 (70.0%) | 22/35 (62.9%) | 17/19 (89.5%) |
|  |  |  |  |  |
|  | Dis=0 | 21/52 (40.4%) | 15/99 (15.2%) | 18/59 (30.5%) |
|  | Dis=1 | 24/40 (60.0%) | 23/69 (33.3%) | 13/23 (56.5%) |
|  | Dis=2 | 27/31 (87.1%) | 30/51 (58.8%) | 4/6 (66.7%) |
|  |  |  |  |  |
| ^a^Age at onset as assessed by original author in schizophrenia samples, and age at first contact with mental health services in psychosis samples. | | | | |

| Table S7. Logistic regression analysis of psychotic syndromes on demographic, developmental and clinical variables in probands in the five twin samples^a^ | | | | | | | | | | |
| --- | --- | --- | --- | --- | --- | --- | --- | --- | --- | --- |
| Demographic, developmental or clinical variable [independent variable] | Psychotic syndrome [dependent variable] | Sample | | | | | | | | |
|  |  | Three schizophrenia samples combined (Fischer, Kringlen, Slater) | | | Maudsley register psychosis sample | | | Maudsley non-register psychosis sample | | |
|  |  | no. probands | OR (95%CI) | p-value | no. probands | OR (95%CI) | p-value | no. probands | OR (95%CI) | p-value |
| Sex (female vs male)  [not adjusted for sex] | Narrow Pos (0-1 vs 2) | 124 | 0.78 (0.35 to 1.74) | 0.536 | 224 | 0.85 (0.48 to 1.52) | 0.585 | 111 | 2.83 (1.13 to 7.08) | 0.027* |
|  | Broad Neg (0 vs 1-2) | 124 | 1.66 (0.75 to 3.66) | 0.208 | 224 | 1.14 (0.66 to 1.95) | 0.636 | 111 | 4.79 (2.08 to 11.04) | 0.00042*** |
|  | Narrow Neg (0-1 vs 2) | 124 | 1.72 (0.81 to 3.67) | 0.159 | 224 | 1.07 (0.51 to 2.24) | 0.861 | 111 | 4.73 (1.74 to 12.87) | 0.003** |
|  | Broad Dis  (0 vs 1-2) | 124 | 1.02 (0.46 to 2.28) | 0.962 | 224 | 1.10 (0.64 to 1.88) | 0.738 | 111 | 0.96 (0.40 to 2.33) | 0.930 |
|  | Narrow Dis (0-1 vs 2) | 124 | 0.61 (0.24 to 1.54) | 0.292 | 224 | 1.16 (0.61 to 2.22) | 0.652 | 111 | None of the 6 probands with Dis=2 were male |  |
|  |  |  |  |  |  |  |  |  |  |  |
| Birth order (first vs second or subsequently born) | Narrow Pos (0-1 vs 2) | 111 | 1.01 (0.43 to 2.33) | 0.989 | 194 | 0.64 (0.34 to 1.20) | 0.162 | 58 | 0.25 (0.03 to 1.90) | 0.173 |
|  | Broad Neg (0 vs 1-2) | 111 | 0.60 (0.25 to 1.41) | 0.237 | 194 | 0.75 (0.42 to 1.32) | 0.318 | 58 | 0.35 (0.10 to 1.25) | 0.102 |
|  | Narrow Neg (0-1 vs 2) | 111 | 0.55 (0.24 to 1.24) | 0.148 | 194 | 0.83 (0.39 to 1.77) | 0.633 | 58 | 0.59 (0.17 to 2.06) | 0.400 |
|  | Broad Dis  (0 vs 1-2) | 111 | 1.04 (0.44 to 2.43) | 0.935 | 194 | 0.65 (0.37 to 1.16) | 0.142 | 58 | 4.12 (1.11 to 15.34) | 0.035* |
|  | Narrow Dis (0-1 vs 2) | 111 | 0.80 (0.31 to 2.04) | 0.629 | 194 | 1.12 (0.58 to 2.16) | 0.740 | 58 | 3.12 (0.55 to 17.71) | 0.194 |
|  |  |  |  |  |  |  |  |  |  |  |
| Age at onset (years)^b^ | Narrow Pos (0-1 vs 2) | 124 | 1.08 (1.03 to 1.15) | 0.004** | 224 | 0.95 (0.92 to 0.97) | 0.000091*** | 111 | 0.98 (0.91 to 1.06) | 0.607 |
|  | Broad Neg (0 vs 1-2) | 124 | 0.96 (0.92 to 1.00) | 0.067 | 224 | 0.96 (0.94 to 0.99) | 0.005** | 111 | 1.02 (0.95 to 1.10) | 0.535 |
|  | Narrow Neg (0-1 vs 2) | 124 | 0.91 (0.86 to 0.96) | 0.00086*** | 224 | 0.94 (0.90 to 0.99) | 0.020* | 111 | 0.99 (0.90 to 1.08) | 0.761 |
|  | Broad Dis  (0 vs 1-2) | 124 | 0.96 (0.92 to 1.01) | 0.130 | 224 | 0.93 (0.90 to 0.96) | 0.000010*** | 111 | 0.99 (0.92 to 1.07) | 0.858 |
|  | Narrow Dis (0-1 vs 2) | 124 | 0.90 (0.84 to 0.96) | 0.002** | 224 | 0.95 (0.91 to 0.99) | 0.010* | 111 | 0.99 (0.90 to 1.09) | 0.815 |
|  |  |  |  |  |  |  |  |  |  |  |
| Illness course (non-chronic vs chronic)^c^ | Narrow Pos (0-1 vs 2) | 122 | 1.82 (0.80 to 4.15) | 0.152 | 219 | 4.98 (2.21 to 11.23) | 0.00013*** | 87 | 20.37 (2.63 to 157.55) | 0.005** |
|  | Broad Neg (0 vs 1-2) | 122 | 2.24 (0.99 to 5.05) | 0.052 | 219 | 4.53 (2.41 to 8.53) | 0.0000044*** | 87 | 24.89 (6.76 to 91.64) | 0.0000062*** |
|  | Narrow Neg (0-1 vs 2) | 122 | 2.10 (0.91 to 4.83) | 0.082 | 219 | 5.36 (2.46 to 11.70) | 0.000033*** | 87 | 28.28 (4.91 to 162.95 | 0.00031*** |
|  | Broad Dis  (0 vs 1-2) | 122 | 4.12 (1.76 to 9.64) | 0.001** | 219 | 4.40 (2.27 to 8.55) | 0.000017*** | 87 | 3.42 (1.27 to 9.22) | 0.016* |
|  | Narrow Dis (0-1 vs 2) | 122 | 7.36 (2.15 to 25.16) | 0.002** | 219 | 5.05 (2.56 to 9.95) | 0.0000048*** | 87 | 5.63 (1.73 to 18.35) | 0.005** |
|  |  |  |  |  |  |  |  |  |  |  |
| ^a^Generalized linear mixed model adjusted for sex and age of co-twin at last information. Additionally, in analysis of the Maudsley register sample, twin pair modelled as a random effect to account for doubly-ascertained pairs and a triplet proband with two co-twins. And in the three schizophrenia samples combined, sample and twin pair modelled as random effects. (OR > 1 indicates association with male sex, being second born, older age at onset, or chronic course.)  ^b^Age at onset as assessed by original author in schizophrenia samples, and age at first contact with mental health services in psychosis samples.  ^c^OPCRIT item 90 score 4 or 5.  *p<0.05, **p<0.01, ***p<0.001, two-tailed. | | | | | | | | | | |

| Table S8. Post hoc sensitivity analysis - Logistic regression analysis of the narrow positive psychotic syndrome on age at onset in probands with DSM-III-R/IV schizophrenia^a^ in the five twin samples^b^ | | | | | | | | | | |
| --- | --- | --- | --- | --- | --- | --- | --- | --- | --- | --- |
| Demographic, developmental or clinical variable [independent variable] | Psychotic syndrome [dependent variable] | Sample | | | | | | | | |
|  |  | Three schizophrenia samples combined (Fischer, Kringlen, Slater) | | | Maudsley register psychosis sample | | | Maudsley non-register psychosis sample | | |
|  |  | no. probands | OR (95%CI) | p-value | no. probands | OR (95%CI) | p-value | no. probands | OR (95%CI) | p-value |
|  |  |  |  |  |  |  |  |  |  |  |
| Age at onset (years)^c^ | Narrow Pos (0-1 vs 2) | 87 | 1.10 (1.02 to 1.19) | 0.020* | 97 | 1.00 (0.89 to 1.13) | 0.969 | 67 | 1.09 (0.88 to 1.34) | 0.439 |
|  |  |  |  |  |  |  |  |  |  |  |
| ^a^DSM-III-R in Maudsley register sample and DSM-IV in the other samples.  ^b^Generalized linear mixed model adjusted for sex and age of co-twin at last information. Additionally, in analysis of the Maudsley register sample, twin pair modelled as a random effect to account for doubly-ascertained pairs and a triplet proband with two co-twins. And in the three schizophrenia samples combined, sample and twin pair modelled as random effects.  ^c^Age at onset as assessed by original author in schizophrenia samples, and age at first contact with mental health services in psychosis samples.  *p<0.05, **p<0.01, ***p<0.001, two-tailed. | | | | | | | | | | |

| Table S9. Probandwise concordance for positive, negative and disorganised psychotic syndromes in the three schizophrenia twin samples individually | | | | | | | |
| --- | --- | --- | --- | --- | --- | --- | --- |
| Phenotypes | | Samples | | | | | |
| Proband phenotype  [present in all probands] | Co-twin phenotype  [absent or present according to concordance] | Fischer schizophrenia sample | | Kringlen schizophrenia sample | | Slater schizophrenia sample | |
|  |  | Concordance | % (95% CI) | Concordance | % (95% CI) | Concordance | % (95% CI) |
|  |  |  |  |  |  |  |  |
| Narrow positive syndrome (Pos=2) | Narrow positive syndrome (Pos=2) | MZ 8/21 | 38.1% (17.3 to 58.9) | MZ 14/43 | 32.6% (18.6 to 46.6) | MZ 12/25 | 48.0% (28.4 to 67.6) |
|  |  |  |  |  |  |  |  |
|  |  |  |  |  |  |  |  |
| Broad negative syndrome (Neg=1 or 2) | Broad negative syndrome (Neg=1 or 2) | MZ 9/18 | 50.0% (26.9 to 73.1) | MZ 18/40 | 45.0% (29.6 to 60.4) | MZ 9/23 | 39.1% (19.2 to 59.1) |
|  |  |  |  |  |  |  |  |
|  |  |  |  |  |  |  |  |
| Narrow negative syndrome (Neg=2) | Narrow negative syndrome (Neg=2) | MZ 4/8 | 50.0% (15.4 to 84.7) | MZ 6/27 | 22.2% (6.5 to 37.9) | MZ 7/16 | 43.8% (19.4 to 68.1) |
|  |  |  |  |  |  |  |  |
|  |  |  |  |  |  |  |  |
| Broad disorganised syndrome (Dis=1 or 2) | Broad disorganised syndrome (Dis=1 or 2) | MZ 3/15 | 20.0% (0.0 to 40.2) | MZ 16/33 | 48.5% (31.4 to 65.5) | MZ 14/24 | 58.3% (38.6 to 78.1) |
|  |  |  |  |  |  |  |  |
|  |  |  |  |  |  |  |  |
| Narrow disorganised syndrome (Dis=2) | Narrow Disorganised syndrome (Dis=2) | MZ 0/3 | 0.0% | MZ 0/9 | 0.0% | MZ 6/19 | 31.6% (10.7 to 52.5) |
|  |  |  |  |  |  |  |  |
|  |  |  |  |  |  |  |  |

| Table S10. Logistic regression analysis - zygosity as a predictor of concordance in the two psychosis twin samples^a^ | | | | | | | |
| --- | --- | --- | --- | --- | --- | --- | --- |
| Phenotypes | | Samples | | | | | |
| Proband phenotype  [analysis confined to pairs where proband had this phenotype] | Co-twin phenotype  [dependent variable -defined as absence or presence of this phenotype] | Maudsley register psychosis sample | | | Maudsley non-register psychosis sample | | |
|  |  | no. probandwise pairs | OR (95%CI) for zygosity as predictor/independent variable (MZ vs DZ) | p-value | no. probandwise pairs | OR (95%CI) for zygosity as predictor/independent variable (MZ vs DZ) | p-value |
|  |  |  |  |  |  |  |  |
| Narrow positive syndrome (Pos=2) | Narrow positive syndrome (Pos=2) | 153 (70 MZ, 85 DZ) | 9.27 (2.97 to 28.91) | 0.00016*** | 77 (61 MZ, 16 DZ) | 9.77 (0.97 to 98.83) | 0.053 |
|  |  |  |  |  |  |  |  |
|  |  |  |  |  |  |  |  |
| Broad negative syndrome (Neg=1 or 2) | Broad negative syndrome (Neg=1 or 2) | 104 (52 MZ, 52 DZ) | 14.45 (1.68 to 123.99) | 0.015* | 51 (41 MZ, 10 DZ) | 12.36 (1.17 to 130.87) | 0.037* |
|  |  |  |  |  |  |  |  |
| Narrow negative syndrome (Neg=2) | Narrow negative syndrome (Neg=2) | n/c - no concordant DZ pairs |  |  | n/c - no concordant DZ pairs |  |  |
|  |  |  |  |  |  |  |  |
| Broad disorganised syndrome (Dis=1 or 2) | Broad disorganised syndrome (Dis=1 or 2) | 122 (57 MZ, 65 DZ) | 16.07 (4.02 to 64.24) | 0.00012*** | n/c - no concordant DZ pairs |  |  |
|  |  |  |  |  |  |  |  |
| Narrow disorganised syndrome (Dis=2) | Narrow Disorganised syndrome (Dis=2) | n/c - no concordant DZ pairs |  |  | n/c - no concordant DZ pairs |  |  |
|  |  |  |  |  |  |  |  |
| n/c, not calculated.  ^a^Generalized linear mixed model: absence or presence of the psychotic syndrome in co-twin as dependent variable, adjusted for sex and age of co-twin at last information. Additionally, in analysis of the Maudsley register sample, twin pair modelled as a random effect to account for doubly-ascertained pairs and a triplet proband with two co-twins. (OR > 1 indicates higher concordance in MZ than DZ pairs.)  *p<0.05, **p<0.01, ***p<0.001, two-tailed. | | | | | | | |

| Table S11. Probandwise concordance for the narrow positive psychotic syndrome (Pos=2) in MZ twin pairs according to the level of negative or disorganised symptoms also occurring in probands | | | | | | | |
| --- | --- | --- | --- | --- | --- | --- | --- |
| Proband phenotype  [present in all probands] | Co-twin phenotype  [absent or present according to concordance] | Three schizophrenia samples combined (Fischer, Kringlen, Slater) | | Maudsley register psychosis sample | | Maudsley non-register psychosis sample | |
|  |  | Concordance | % | Concordance | % | Concordance | % |
|  |  |  |  |  |  |  |  |
| Narrow positive syndrome (Pos=2) | Narrow positive syndrome (Pos=2)  [for all analyses] |  |  |  |  |  |  |
| and: |  |  |  |  |  |  |  |
| Negative syndrome = 0 (Neg=0) |  | MZ 10/35 | 28.6% | MZ 11/28 | 39.3% | MZ 8/22 | 36.4% |
|  |  |  |  |  |  |  |  |
|  |  |  |  |  |  |  |  |
| Negative syndrome = 1 (Neg=1) |  | MZ 9/22 | 40.9% | MZ 13/31 | 41.9% | MZ 5/18 | 27.8% |
|  |  |  |  |  |  |  |  |
|  |  |  |  |  |  |  |  |
| Negative syndrome = 2 (Neg=2) |  | MZ 15/32 | 46.9% | MZ 5/11 | 45.5% | MZ 10/21 | 47.6% |
|  |  |  |  |  |  |  |  |
|  |  |  |  |  |  |  |  |
|  |  |  |  |  |  |  |  |
| Disorganised syndrome = 0 (Dis=0) |  | MZ 9/36 | 25.0% | MZ 2/21 | 9.5% | MZ 12/39 | 30.8% |
|  |  |  |  |  |  |  |  |
|  |  |  |  |  |  |  |  |
| Disorganised syndrome = 1 (Dis=1) |  | MZ 14/32 | 43.8% | MZ 15/27 | 55.6% | MZ 10/17 | 58.8% |
|  |  |  |  |  |  |  |  |
|  |  |  |  |  |  |  |  |
| Disorganised syndrome = 2 (Dis=2) |  | MZ 11/21 | 52.4% | MZ 12/22 | 54.5% | MZ 1/5 | 20.0% |
|  |  |  |  |  |  |  |  |

| Table S12. Probandwise concordance for narrow positive sub-syndromes in the five twin samples | | | | | | | |
| --- | --- | --- | --- | --- | --- | --- | --- |
| Proband phenotype  [present in all probands] | Co-twin phenotype  [absent or present according to concordance] | Three schizophrenia samples combined (Fischer, Kringlen, Slater) | | Maudsley register psychosis sample | | Maudsley non-register psychosis sample | |
|  |  | Concordance | % (95% CI) | Concordance | % (95% CI) | Concordance | % (95% CI) |
| Pos=2 and: | Pos=2 and: |  |  |  |  |  |  |
| Neg=0 | Neg=0 | MZ 5/35 | 14.3% (2.7 to 25.9) | MZ 7/28 | 25.0% (9.0 to 41.0) | MZ 4/22 | 18.2% (2.1 to 34.3) |
|  |  |  |  | DZ 0/34 | 0.0% | DZ 0/7 | 0.0% |
|  |  |  |  |  |  |  |  |
| Neg=1or2 | Neg=1or2 | MZ 18/54 | 33.3% (20.8 to 45.9) | MZ 12/42 | 28.6% (14.9 to 42.2) | MZ 12/39 | 30.8% (16.3 to 45.3) |
|  |  |  |  | DZ 1/49 | 2.0% (0.0 to 6.0) | DZ 0/9 | 0.0% |
|  |  |  |  |  |  |  |  |
| Neg=2 | Neg=2 | MZ 6/32 | 18.8% (5.2 to 32.3) | MZ 2/11 | 18.2% (0.0 to 41.0) | MZ 1/21 | 4.8% (0.0 to 13.9) |
|  |  |  |  | DZ 0/21 | 0.0% | DZ 0/2 | 0.0% |
|  |  |  |  |  |  |  |  |
| Dis=0 | Dis=0 | MZ 6/36 | 16.7% (4.5 to 28.8) | MZ 2/21 | 9.5% (0.0 to 22.1) | MZ 10/39 | 25.6% (11.9 to 39.3) |
|  |  |  |  | DZ 2/28 | 7.1% (0.0 to 16.7) | DZ 1/11 | 9.1% (0.0 to 26.1) |
|  |  |  |  |  |  |  |  |
| Dis=1or2 | Dis=1or2 | MZ 19/53 | 35.8% (22.9 to 48.8) | MZ 27/49 | 55.1% (41.2 to 69.0) | MZ 3/22 | 13.6% (0.0 to 28.0) |
|  |  |  |  | DZ 3/55 | 5.5% (0.0 to 11.5) | DZ 0/5 | 0.0% |
|  |  |  |  |  |  |  |  |
| Dis=2 | Dis=2 | MZ 3/21 | 14.3% (0.0 to 29.3) | MZ 7/22 | 31.8% (12.4 to 51.3) | MZ 0/5 | 0.0% |
|  |  |  |  | DZ 0/24 | 0.0% | DZ 0/1 | 0.0% |
|  |  |  |  |  |  |  |  |

| Table S13. Sensitivity analysis - probandwise concordance for the psychotic syndromes and narrow positive sub-syndromes in MZ^a^ twins with DSM-III-R/IV schizophrenia^b^ in the five twin samples | | | | | | | |
| --- | --- | --- | --- | --- | --- | --- | --- |
| Proband phenotype  [present in all probands] | Co-twin phenotype  [absent or present according to concordance] | Three schizophrenia samples combined (Fischer, Kringlen, Slater) | | Maudsley register psychosis sample | | Maudsley non-register psychosis sample | |
|  |  | Concordance | % (95% CI) | Concordance | % (95% CI) | Concordance | % (95% CI) |
| DSM Sz and: | DSM Sz and: |  |  |  |  |  |  |
|  |  |  |  |  |  |  |  |
| Main syndromes |  |  |  |  |  |  |  |
| Pos=2 | Pos=2 | 22/68 | 32.4% (21.2 to 43.5) | 19/45 | 42.2% (27.8 to 56.7) | 18/50 | 36.0% (22.7 to 49.3) |
| Neg=1or2 | Neg=1or2 | 23/61 | 37.7% (25.5 to 49.9) | 8/33 | 24.2% (9.6 to 38.9) | 17/40 | 42.5% (27.2 to 57.8) |
| Neg=2 | Neg=2 | 8/38 | 21.1% (8.1 to 34.0) | 2/9 | 22.2% (0.0 to 49.4) | 3/21 | 14.3% (0.0 to 29.3) |
| Dis=1or2 | Dis=1or2 | 21/54 | 38.9% (25.9 to 51.9) | 20/39 | 51.3% (35.6 to 67.0) | 5/22 | 22.7% (5.2 to 40.2) |
| Dis=2 | Dis=2 | 5/24 | 20.8% (4.6 to 37.1) | 7/19 | 36.8% (15.2 to 58.5) | 2/5 | 40.0% (0.0 to 82.9) |
|  |  |  |  |  |  |  |  |
| Sub-syndromes of Pos=2 |  |  |  |  |  |  |  |
| Pos=2 & Neg=0 | Pos=2 & Neg=0 | 2/23 | 8.7% (0.0 to 20.2) | 3/13 | 23.1% (0.2 to 46.0) | 2/11 | 18.2% (0.0 to 41.0) |
| Pos=2 & Neg=1or2 | Pos=2 & Neg=1or2 | 13/45 | 28.9% (15.7 to 42.1) | 8/32 | 25.0% (10.0 to 40.0) | 11/39 | 28.2% (14.1 to 42.3) |
| Pos=2 & Neg=2 | Pos=2 & Neg=2 | 3/27 | 11.1% (0.0 to 23.0) | 2/9 | 22.2% (0.0 to 49.4) | 1/21 | 4.8% (0.0 to 13.9) |
|  |  |  |  |  |  |  |  |
| Pos=2 & Dis=0 | Pos=2 & Dis=0 | 4/25 | 16.0% (1.6 to 30.4) | 0/8 | 0.0% | 8/29 | 27.6% (11.3 to 43.9) |
| Pos=2 & Dis=1or2 | Pos=2 & Dis=1or2 | 14/43 | 32.6% (18.6 to 46.6) | 19/37 | 51.4% (35.3 to 67.5) | 2/21 | 9.5% (0.0 to 22.1) |
| Pos=2 & Dis=2 | Pos=2 & Dis=2 | 3/16 | 18.8% (0.0 to 37.9) | 7/17 | 41.2% (17.8 to 64.6) | 0/5 | 0.0% |
|  |  |  |  |  |  |  |  |
| ^a^No DZ pairs concordant for DSM-III-R/IV schizophrenia (psychosis samples), or insufficient information available on DZ pairs (schizophrenia samples).  ^b^DSM-III-R in Maudsley register sample and DSM-IV in the other samples. | | | | | | | |

| Table S14. Sensitivity analysis - probandwise concordance for the psychotic syndromes and narrow positive sub-syndromes in MZ^a^ twins with DSM-III-R/IV schizophrenia^b^ in the four systematically-ascertained samples combined, and in all five twin samples combined | | | | | |
| --- | --- | --- | --- | --- | --- |
| Proband phenotype  [present in all probands] | Co-twin phenotype  [absent or present according to concordance] | The four systematically-ascertained samples combined (Fischer, Kringlen, Slater, Maudsley register) | | All five samples combined (Fischer, Kringlen, Slater, Maudsley register, Maudsley non-register) | |
|  |  | Concordance | % (95% CI) | Concordance | % (95% CI) |
| DSM Sz and: | DSM Sz and: |  |  |  |  |
|  |  |  |  |  |  |
| Main syndromes |  |  |  |  |  |
| Pos=2 | Pos=2 | 41/113 | 36.3% (27.4 to 45.2) | 59/163 | 36.2% (28.8 to 43.6) |
| Neg=1or2 | Neg=1or2 | 31/94 | 33.0% (23.5 to 42.5) | 48/134 | 35.8% (27.7 to 43.9) |
| Neg=2 | Neg=2 | 10/47 | 21.3% (9.6 to 33.0) | 13/68 | 19.1% (9.8 to 28.5) |
| Dis=1or2 | Dis=1or2 | 41/93 | 44.1% (34.0 to 54.2) | 46/115 | 40.0% (31.1 to 49.0) |
| Dis=2 | Dis=2 | 12/43 | 27.9% (14.5 to 41.3) | 14/48 | 29.2% (16.3 to 42.0) |
|  |  |  |  |  |  |
| Sub-syndromes of Pos=2 |  |  |  |  |  |
| Pos=2 & Neg=0 | Pos=2 & Neg=0 | 5/36 | 13.9% (2.6 to 25.2) | 7/47 | 14.9% (4.7 to 25.1) |
| Pos=2 & Neg=1or2 | Pos=2 & Neg=1or2 | 21/77 | 27.3% (17.3 to 37.2) | 32/116 | 27.6% (19.5 to 35.7) |
| Pos=2 & Neg=2 | Pos=2 & Neg=2 | 5/36 | 13.9% (2.6 to 25.2) | 6/57 | 10.5% (2.6 to 18.5) |
|  |  |  |  |  |  |
| Pos=2 & Dis=0 | Pos=2 & Dis=0 | 4/33 | 12.1% (1.0 to 23.3) | 12/62 | 19.4% (9.5 to 29.2) |
| Pos=2 & Dis=1or2 | Pos=2 & Dis=1or2 | 33/80 | 41.3% (30.5 to 52.0) | 35/101 | 34.7% (25.4 to 43.9) |
| Pos=2 & Dis=2 | Pos=2 & Dis=2 | 10/33 | 30.3% (14.6 to 46.0) | 10/38 | 26.3% (12.3 to 40.3) |
|  |  |  |  |  |  |
| ^a^No DZ pairs concordant for DSM-III-R/IV schizophrenia (psychosis samples), or insufficient information available on DZ pairs (schizophrenia samples).  ^b^DSM-III-R in Maudsley register sample and DSM-IV in the other samples. | | | | | |
